# Design, Development, and Analysis of an Automated Sampling Loop for Online Monitoring of Chiral Crystallization

**DOI:** 10.1021/acs.oprd.1c00320

**Published:** 2022-02-15

**Authors:** Ghufran ur Rehman, Thomas Vetter, Philip A. Martin

**Affiliations:** Department of Chemical Engineering and Analytical Science, University of Manchester, M13 9PL Manchester, U.K.

**Keywords:** online monitoring, automated sampling, chiral crystallization, PAT, polarimeter

## Abstract

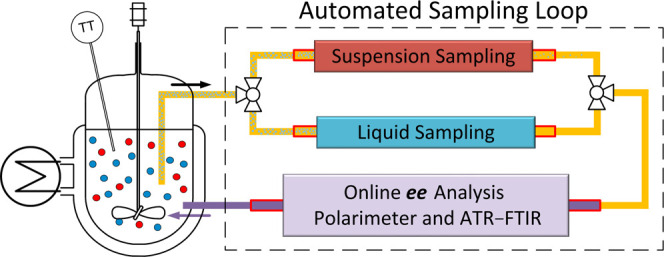

Enantiomeric purity is of prime importance for several industries, specifically in the production of pharmaceuticals. Crystallization processes can be used to obtain pure enantiomers in a suitable solid form. However, some process variants inherently rely on kinetic enhancement (preferential crystallization) of the desired enantiomer or on complex interactions of several phenomena (e.g., attrition-enhanced deracemization and Viedma ripening). Thus, a process analytical technology able to measure the enantiomeric composition of both the solid phase and the liquid phase would be valuable to track and eventually control such processes. This study presents the design and development of a novel automated analytical monitoring system that achieves this. The designed setup tracks the enantiomeric excess (*ee*) using a continuous closed-loop sampling loop that is coupled to a polarimeter and an attenuated total reflection Fourier transform infrared spectroscopy spectrometer. By heating the loop and alternately sampling either the liquid or the suspension, the combination of these measurements allows tracking of the *ee* of both the liquid and the solid. This work demonstrates a proof of concept of both the experimental and theoretical aspects of the new system.

## 1 Introduction

Purity in chiral compounds is of increasing importance for the chemical industries. In the case of pharmaceuticals, there is ample evidence for several drugs showing that only one enantiomer provides the required physiological effect. The other enantiomer often has no biological effect or is even toxic when ingested.^1−3^ Some examples of chiral compounds associated with toxic responses are ethambutol,^4^ ketamine,^5^ thalidomide,^6,7^ and benoxaprofen.^8^ Various cases such as these led to the U.S. Food and Drug Administration (FDA) demanding that chiral drugs are produced in their optically purest form, having only one enantiomer in the product.^9^ This requirement has led to extensive research to find new and improved ways for obtaining enantiomerically pure drugs. In recent decades, most of the active pharmaceutical ingredients (APIs) are now being manufactured in the enantiopure form.^10,11^

Crystallization is one of the key unit operations in this context. Its widespread and common use is due to the fact that purification and separation processes can be executed in a single batch or continuous operation with high purity.^12−14^ Even so, there still remain a number of challenges linked to its design, stability, and operational control, which affect the final properties of the product such as purity, size, or particle shape.^15,16^ These properties in turn can strongly affect the operation of downstream processes, such as filtration,^17^ drying,^18^ and milling.^19^

One way to minimize these issues is by using relevant characterization techniques to either create deeper process understanding and then adapting the recipe of the process or by applying them for (feedback) control purposes. The introduction of the PAT (process analytical technology) initiative in 2004 has led to a wide range of new on-line and in-line analytical techniques being applied to pharmaceutical processing and crystallization in particular.^20−22^ Among those of relevance to crystallization are attenuated total reflection Fourier transform infrared spectroscopy (ATR–FTIR) and ultraviolet—visible absorption spectroscopy (ATR–UV/vis), Raman spectroscopy, focused beam reflectance measurement (FBRM), particle vision and measurement (PVM), and chiral chromatography. ATR–FTIR and ATR–UV/vis are used to determine the concentration of species from the absorption spectrum often using advanced multivariate analysis techniques such as partial least-squares regression (PLSR) (as used in this work).^23,24^ Raman spectroscopy can also measure species concentrations including enantiomers^25^ as well as determine polymorphism. Particle size distributions (PSDs) can be determined using FBRM^26^ and PVM.^27^ In FBRM, chord lengths and chord length distributions can be determined on-line and these can then be used to derive PSDs. PVM is based on an on-line video image of the evolving particles in the process, which can again be used to derive particle or crystal size distributions and morphologies. On-line chiral chromatography^28,29^ can be used to separate and identify different optical isomers or enantiomers by HPLC (high-performance liquid chromatography). Polarimeters (as used in this work) can be used for determining the presence and concentration of enantiomers by measuring the angle of rotation of linearly polarized light when passed through the sample.^30^

By utilizing the data obtained directly from process measurements, near-real-time control^31,32^ and scientific understanding of how process parameters affect product quality and performance are enabled.^33^ Our design is focused on utilizing this approach for the monitoring and control of chiral resolution processes.

Chiral crystallization (chiral resolution) is a process for separation of pure enantiomers from a racemic mixture. The specific design of this process depends strongly on the type of the solid phase and its phase diagram. Enantiomers can crystallize either as a racemic compound (equal ratio of both enantiomers in a structured crystalline array), as a conglomerate (physical mixture of enantiomerically pure crystals), or as a solid solution (<2% of crystals formed).^13,34,35^ Apart from classical diastereomeric resolutions, chiral resolution processes are mostly used for resolving conglomerates as the enantiomers are easily separable. Racemic compounds are more common (89%) but are very challenging to separate by crystallization: they can be separated only if there is sufficient enrichment in the feed.^16^ Out of various chiral resolution methods, preferential crystallization (PC) and Viedma ripening (VR) processes provide an effective way to achieve conglomerate separations.^13,36,37^

**Figure 1:**
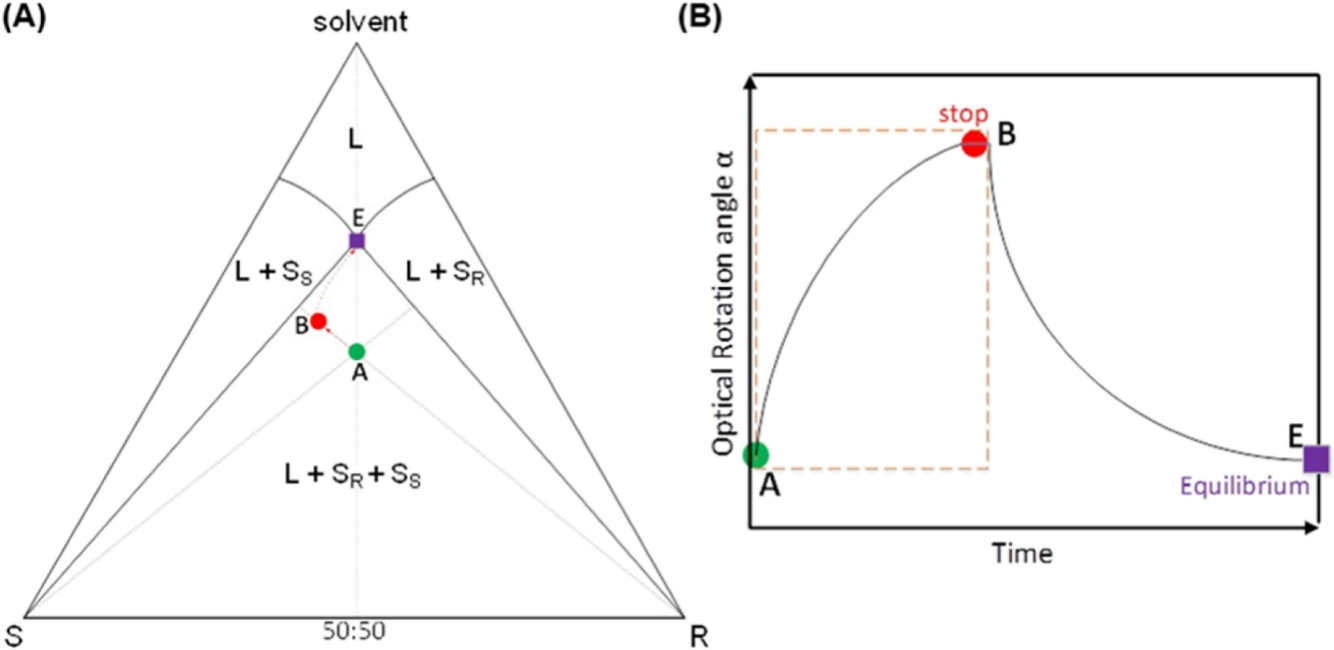
PC: (A) PC conglomerate phase diagram, where R and S refer to enantiomers, L represents liquid, and S_R_ and S_S_ refer to pure solid. PC is initiated by adding seed crystals at point A that subsequently grow. The process eventually reaches its thermodynamic equilibrium in point E and needs to be stopped in point B before nucleation and growth of crystals of the undesired enantiomer occur. (B) Anticipated absolute value of the optical rotation angle (alpha-α) in the liquid phase during PC.

PC is a stereo-selective process, in which the desired enantiomer is selectively crystallized from a racemic supersaturated solution. The desired enantiomer is kinetically enhanced by the addition of seed crystals of this enantiomer. However, the process will eventually approach its thermodynamic equilibrium (which is a racemic mixture in the solid and the liquid), that is, crystals of the undesired enantiomer will nucleate and subsequently grow. Stopping the process by filtering the enriched solid phase at the right time is critical (Figure 1). Stopping early is robust but can also result in sacrificing conversion into the desired enantiomer, which in return causes loss of productivity. However, stopping the process too late can cause precipitation of the supersaturated undesired enantiomer that results in loss of purity. For this case, tracking the liquid phase composition allows identification of the point to stop and prevent forming crystals of the undesired enantiomer.

**Figure 2:**
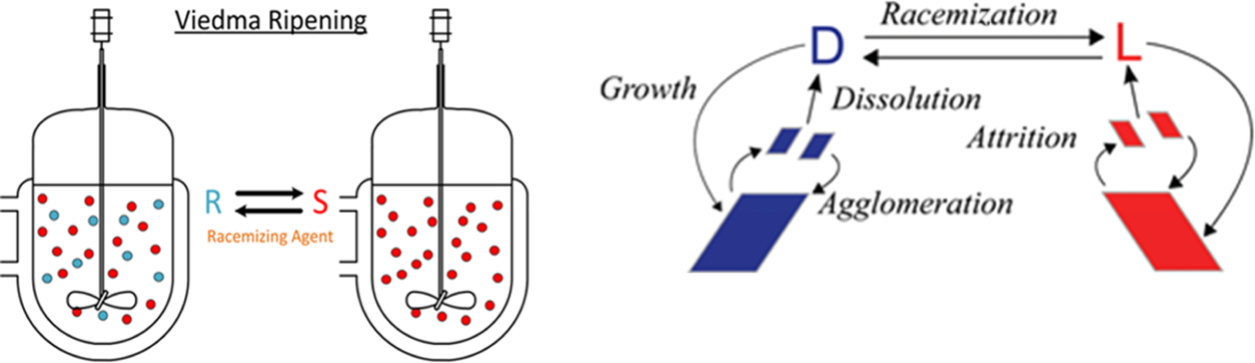
VR (left) the result of the process is that a suspension containing crystals of both the R and S enantiomers get converted to crystals of only the S enantiomer in the presence of a liquid-phase racemization agent/catalyst and (right) depiction of the mechanisms involved (see refs (47) and (48) for an in-depth explanation of mechanistic details).^49^

VR, as shown in Figure 2, involves grinding of a crystal mixture with an initial enantiomeric excess (*ee*) under saturated conditions; the solid-state processes and a liquid-phase racemization reaction lead to deracemization of the solid phase.^38,39^ This process strongly depends on the initial conditions and is affected by various factors such as attrition, agglomeration, growth, and dissolution on the solid phase side and the racemization kinetics occurring in the liquid phase.^40,41^ In this process, the liquid composition remains (nearly) the same but the solid phase enantiomeric excess increases over time. Tracking the solid phase composition provides information on progress of the process and is useful to know when completion is attained at 100% *ee*. In recent years, extensive research has been carried out on thermal variants of such processes with a racemization reaction in solution, specifically on temperature cycling,^42−45^ as a replacement mechanism for grinding. Since the change of temperature induces crystal dissolution and growth to a stronger degree than that in isothermal VR processes, it becomes instructive to track the solution composition and the solid phase composition in terms of enantiomeric excess at the same time. Measuring the *ee* of the liquid phase has been previously demonstrated for the case of PC processes,^30,34^ but for processes such as VR, the analysis is still carried out by manual sampling.^41,46^

In this contribution, our main aim is therefore to introduce the design of an efficient automated system that enables real-time characterization of the enantiomeric excess (*ee*) for the solid as well as liquid phases in chiral resolution processes. The system introduced here achieves this by alternately sampling the mother liquor and the suspension (i.e., mother liquor plus crystals). The former is accomplished by sending the sampled stream through a filter, while in the latter measurement mode, the filter system is bypassed. In both modes, the sampled stream (after the filter system) is heated so that all crystals are dissolved and also to relieve supersaturation and avoid nucleation when the filter is used and the supernatant is sampled. In order to measure the difference in enantiomer concentration in the sampled stream,^30,50^ the rotation angle can, for example, be measured using a polarimeter, whereas to measure the sum of concentrations of both enantiomers as well as to detect the presence of any impurities,^51,52^ an infrared spectrometer with an ATR probe can be used (ATR–FTIR). In the liquid sampling mode, the two measurements directly allow calculation of the liquid phase enantiomeric excess. We will show that the solid phase enantiomeric excess can be calculated using a combination of the liquid phase and the suspension mode measurements.

In order to operate such a measurement device continuously and in an automated fashion, several challenges had to be addressed such as automated clearing of the filter system, ensuring representative sampling of the crystallizer suspension, and establishing appropriate sampling loop heating and flow rates to ensure dissolution of the solid, as well as assessing inaccuracies caused by the continuing racemization reaction while sampling (in the case of VR processes).

In this work, we explore an experimental proof of concept for an automated sampling setup and the principles of both operating modes: the liquid and suspension sampling modes. In the latter section, we also show that the auto-switch cyclic mechanism and the declogging of the filter system can result in a continuous stable and steady suspension sampling operation. This is combined with a modeling study elucidating the operating range of the measurement device in terms of temperatures, flow rates, and the underlying kinetics of dissolution. The operating parameters of the sampling loop setup may be considered by using energy balances for the heated tube segments and the crystallizer, the population balance equations (PBEs) and the mass balance equations (MBEs).

## 2 Materials and Methods

### 2.1 Chemicals

l-Asparagine monohydrate (L-AM) of purity 98% was purchased from Affymetrix, isopropanol (IPA) of 99.5% purity was obtained from Sigma Aldrich, and water of Type 1 from Milli-Q Ultrapure Water was used as a solvent with 2–3 wt % IPA. We found the latter to be useful to prevent/slow down the growth of microorganisms in saturated solutions that were kept for multiple runs. Solubility data of L-AM were obtained from previous studies.^53^ These data were utilized for making saturated solutions and verified via gravimetric analysis for each batch.

### 2.2 Automated Sampling Loop Setup

**Figure 3:**
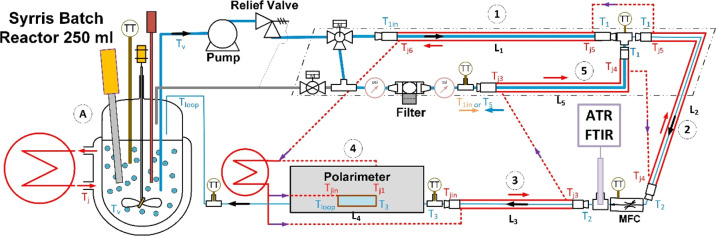
Schematic of the automated continuous sampling setup for online monitoring of a chiral crystallization process. The setup consists of a crystallizer and an automated sampling loop; both are equipped with a thermostat to allow for independent temperature control. The sampling loop consists of a pump, a filter system, heated tube segments (1–5, tube-in-tube configuration), temperature and pressure transmitters (TT, psi), and a mass flow controller. Measurements are obtained using an ATR–FTIR spectrometer and a polarimeter.

A schematic of the continuous sampling setup is shown in Figure 3. Crystallization was performed in an Atlas automated 250 mL jacketed reactor system (Atlas Potassium Setup, Syriss Ltd, Cambridge UK). The Atlas system consisted of an integrated overhead stirrer, a temperature probe, a turbidity probe, and thermostat 1 (Huber Ministat 230cc, Offenburg, Germany) synchronized using Syriss software for direct monitoring and control of the crystallizer. The thermostat liquid used was silicone oil (SIL 180) with a temperature range from −20 to +200 °C. A dispersing tool (Ultra Turax T25, IKA Germany) was installed in the reactor vessel for crushing crystals into smaller particles (30–50 μm) and to avoid formation of large agglomerates. As is detailed below, the addition of the dispersing tool was necessary to enable representative sampling of the suspension. The dispersing tool also enhances mixing in the crystallizer.^45^ The solution mixture was taken directly from the reactor vessel through a 1/4 in. PFA transparent tube via a pump (Watson Marlow Qdos 30 ReNu Peristaltic Pump, Manchester UK) that is equipped with a pressure relief valve (Swagelok, Manchester UK). When triggered (caused by a blockage somewhere in the loop), the pumped fluid was diverted back to the crystallizer, and the operation of the system was stopped. The relief valve was followed by an electrically actuated three-way and two-way valve (Swagelok, Manchester UK), which allows operation of the loop in two modes: liquid or suspension sampling, which are run alternately in a continuous closed loop as required. These modes of operation were applied either with fixed time intervals (chosen depending on the kinetics of the process to be monitored) or with an automatic switching mechanism that was triggered according to the pressure drop measured across the filter system. The two sampling modes are detailed in the following sections. The sampling loop itself consists of heated tube segments (tube-in-tube heat exchangers). In order to strike a balance between the ability to let particles flow through the tubes, residence time, and volume of fluid in the sampling loop, segments 1 and 5 consist of 1/4 in. PFA tubing, while segments 2 and 3 consist of 1/8 inch PFA tubing; segment 4 is the polarimeter measurement cell, which is also jacketed and is made from stainless steel.

**Figure 4:**
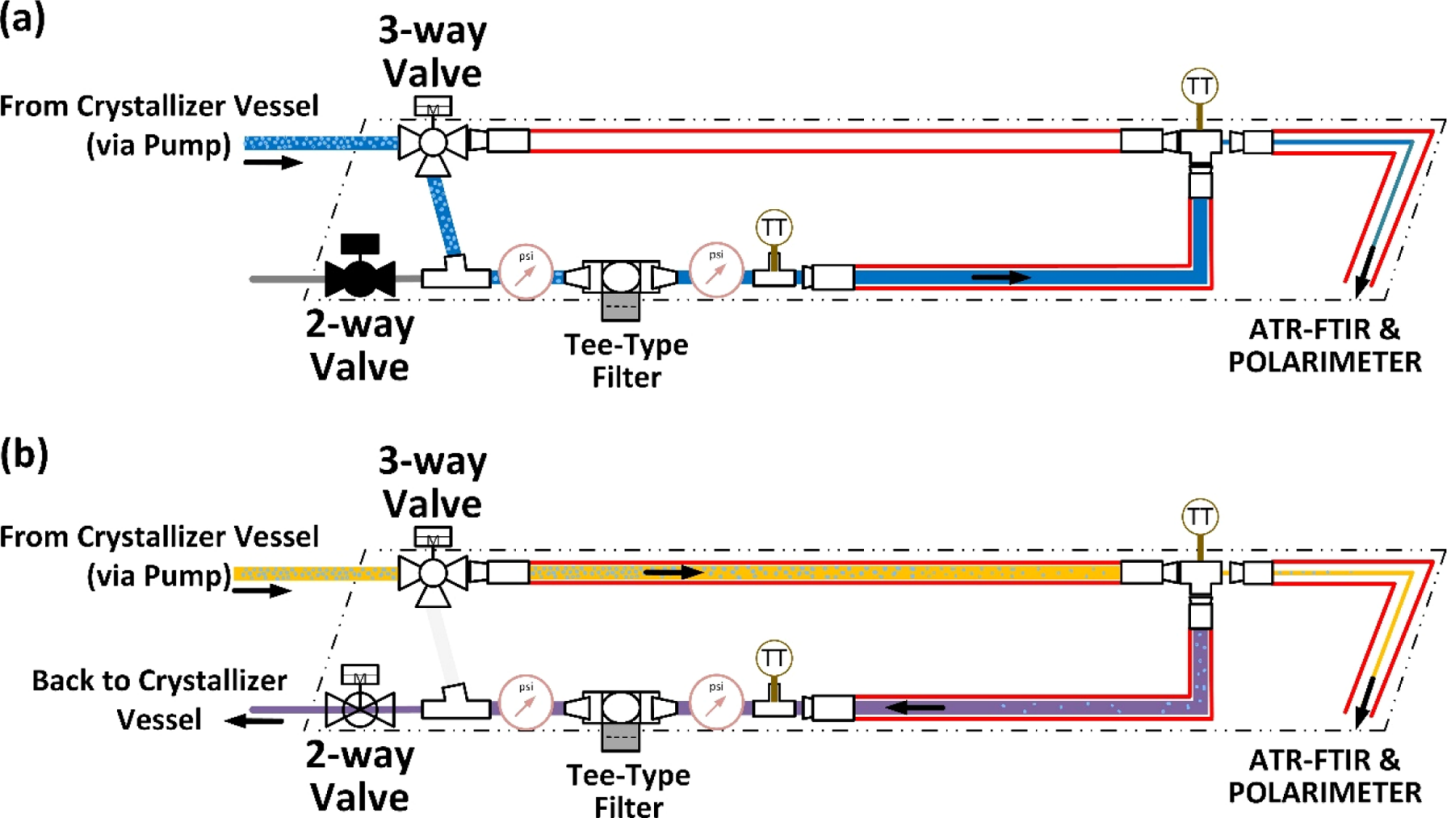
Process flow diagram of continuous sampling mode of operations. (a) Liquid sample: filtration of the solid crystals to analyze the liquid phase. (b) Suspension sampling: analysis of the solid phase by dissolution of particles using tube-in-tube heating and back-purge of solid-free stream for filter cleaning. Both modes are engaged alternatingly for determining solid *ee*.

Liquid sampling was achieved using a filtration loop, as shown in Figure 4a. In this loop, the crystal mixture was filtered using a stainless-steel filter with a 0.5 μm pore size (Swagelok, Manchester UK). The solid particles were filtered out, and the solution was passed through for liquid phase analysis. Note that when monitoring a crystallization process, the solution is supersaturated at this point, which has the potential to clog the system downstream. However, due to the heated tube segments and the heated polarimeter measurement cell [i.e., the segments labeled with 5, 2, 3, and 4 (in the order of flow) in Figure 3], the solution quickly reaches undersaturated conditions. Note that segments 2 and 3 are operated in counter-current flow, while segment 5 is operated in co-current flow in this sampling mode. Note also that the automated two-way valve was kept in the closed position in this operating mode. Pressure transducers (Swagelok, Manchester UK) were placed on both sides of the filter to check the pressure drop across it, which sharply increases during the onset of clogging. The time until this increase in pressure drop is reached depends on a variety of factors including the suspension density of the crystallizer (higher suspension density leading to faster clogging), the growth rate of the crystals under the current conditions in the crystallizer (faster growth rate leading to faster clogging), and substance-specific properties. Therefore, the pressure drop measured across the filter was used to trigger automatic switching to the suspension sampling mode (the threshold for this was set at 1.5 bar). As is explained below, the filter system can be back-purged with heated, undersaturated solution during suspension sampling. This represents an effective and automated cleaning mechanism for the filter system, which enables long-term continuous operation of the sampling loop without manual interventions.

Suspension sampling was operated using a filter bypass setup using a three-way valve, as shown in Figure 4b. The sampled suspension required heating to ensure that no particles reach the measurement devices in the loop. Failing to ensure this will lead the polarimeter to report erroneous values and the mass flow controller (MFC) to potentially clog. Therefore, also in this sampling mode, the tube-in-tube heat exchanger setup performs a vital role. As evident from Figure 3, the flow path of a suspension sample is through loop segments 1, 2, 3, and 4 (all operated as counter-current heat exchangers). The conditions along this flow path should be chosen so that all particles are dissolved before the MFC (Figure 3) is reached.

In the suspension sampling mode, part of the sampled stream exiting loop segment 1 can be diverted through loop segment 5 and pushed across the filter system (unless it is completely clogged; the previously defined 1.5 bar pressure drop was found to be a conservative, i.e., pre-clogging, value). The automated two-way valve was in an open position in this operating mode. Clogged crystals were purged back into the crystallizer (preventing wastage of the crystal product), which were detected with the aid of the turbidity probe. The combination of the control valve and pump was used to maintain the back-purge flow. The flow rate through the polarimeter was usually kept between 50 and 55 mL/min for both modes using an MFC (Mini CORI-FLOW M mass flow meter equipped with a F-004AC valve, Bronkhorst NL). During liquid sampling, the flow was controlled by the MFC acting on the peristaltic pump directly, while the control valve on the MFC was kept in a fully open position. Conversely, in the suspension sampling mode, the pump was set to a higher, constant (usually nominally double) pump rate, while the control valve on the MFC acted to keep the flow rate through the MFC constant. This meant that the excess flow generated by the pump was diverted through tube segment 5 and across the filter system; in turn back-purging and cleaning it. Note that the tube-in-tube heat exchanger in segment 5 operated in the counter-current mode (it operates in the co-current mode during liquid sampling) to guarantee efficient heat transfer. This was deemed necessary because the flow path for the back-purge stream is shorter than that for the suspension sampling stream, while the back-purge stream ideally also reaches undersaturated conditions to efficiently clean the filter system.

The temperature of the heated loop was tracked along the sampling loop using RTD probes (OMEGATM UK), as indicated (TT) in Figure 3. The temperature of thermostat 2 (containing water as a thermofluid) was controlled through the RTD probe located before the polarimeter sampling cell. The set point was chosen to be high enough to guarantee undersaturated conditions before the MFC and in the back-purge stream (which was also evaluated using the model detailed below). Note that the flow out of thermostat 2 was split in two (roughly equal) parts, one entering directly into the jacket of segment 3 (and then further through the jackets of tube segments 5, 2, and 1 before returning to the thermostat), while the other one was used to heat the polarimeter sampling cell (tube segment 4).

The overall volume of the sample solution drawn into the loop was designed to be no more than 50–60 mL (depending on the mode of operation). Automated process control of the sampling loop setup was constructed using an Arduino microcontroller, a Siemens adapter, DAQ, and PT-104 hardware. The batch reactor vessel was controlled using the Atlas Software system to enable complex recipe control and real-time data plotting. The control box graphical user interface (GUI) was built using LabVIEW, as shown in the Supporting Information (Section 2.7). It provides a user-friendly interface for monitoring and control of valve operation modes, sample flow rate, and temperature set point values and displays real-time measurement of optical rotation, temperature gradient in the loop, and pressure drop across the filter.

### 2.3 Measurement Methods

The setup was equipped with two analytical instruments that were connected in-line in the sampling loop. The first was an ATR–FTIR system (ReactIR-45m ATR–FTIR, Mettler Toledo Germany) equipped with an AgX 9.5 mm DiComp ATR probe. It has a penetration depth of 2 μm and was used to monitor the total concentration of enantiomers in the liquid phase. Spectra were collected at 1 min intervals and averaged over 128 scans. A spectral resolution of 8 cm^–1^ was used across the range 650–1900 cm^–1^. Calibration was required for relating the measured variables (absorbance peak height) with the independent variable (total concentration). In the liquid phase, factors such as composition, temperature, and viscosity can influence the IR absorbance; therefore, multivariate data analysis was applied in order to obtain more accurate and robust results.^51,52^ PLSR was used for the concentration calibration model,^52^ and the details of the method are described in the Supporting Information.

The polarimeter (POLARmonitor, IBZ Messtechnik GmbH Germany) was equipped with an LED light source (589 nm) and a jacketed SS 316L measurement flow cell of 10 cm path length for continuous tracking of the concentration difference between the enantiomers. Constant flow rate and temperature were required for optimal measurement. Zero baseline correction was performed using a pure solvent before and after each experiment. The combined data obtained from these tools enable us to determine the enantiomeric excess of either the liquid or the (dissolved) suspension, depending on the sampling mode. Each optically active molecule was defined in terms of its specific rotation [α]_λ_^T^. This was first described in Biot’s law,^54^ which states that
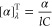
1where α is the observed optical rotation [deg] at a specific temperature and wavelength, *l* is the path length of the measuring cell [d*m*], and *C* is the concentration [g/mL]. Furthermore, two enantiomers of the same substance exhibit a specific rotation with the same absolute value but opposite sign.^30,54^ This means that for mixtures of the two enantiomers, α is proportional to the difference in concentration between the R and S enantiomers. The ATR–FTIR analysis in turn cannot distinguish between different enantiomers. However, we can obtain the total concentration of enantiomers (*C*_S_ + *C*_R_) and can therefore determine the enantiomeric excess from *ee* = (*C*_S_ – *C*_R_)/(*C*_S_ + *C*_R_). Using both liquid and suspension sampling modes of operation, as explained in Section 2.2, we can therefore obtain the enantiomeric excess for the liquid and the dissolved suspension (dsus) (both given in eq 2). The solid enantiomeric excess *ee*_solid_ was therefore determined by evaluating the difference of each measurement in the liquid (l) and the dissolved suspension mixture given by eq 3, which is valid when there are no additional chiral impurities present
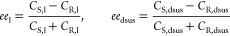
2
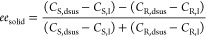
3

**Figure 5:**
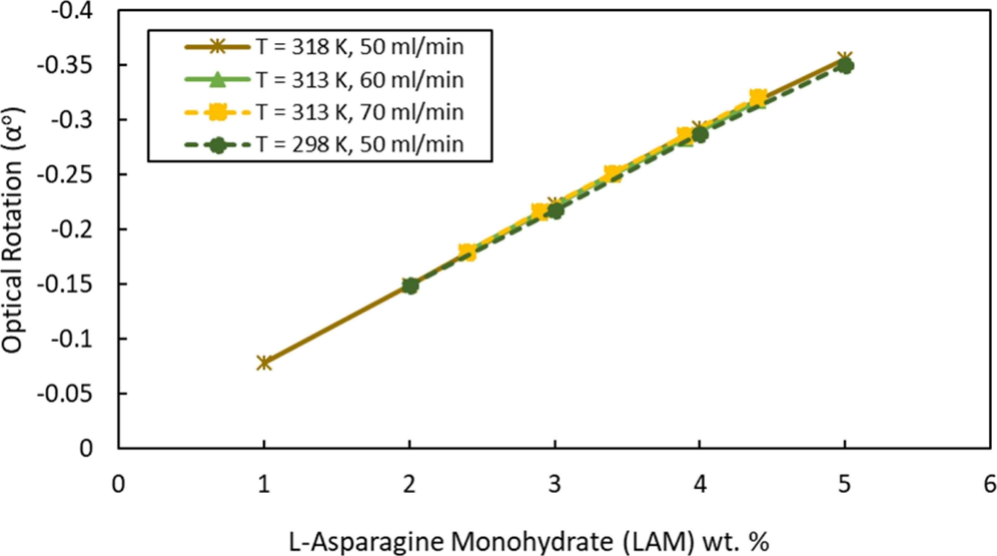
L-AM calibration experiment for the polarimeter (data points: measured alpha values at variable flow rates and loop temperature and lines: linear fitting).

Polarimeter calibration was achieved by measuring pure L-AM of different concentrations (covering a range between diluted to supersaturated solution) dissolved in deionized water by continuous sampling in the loop setup. During measurement, the flow rate and temperature were kept constant. Variation of these parameters induced mechanical stress on the measuring cell windows, which could create some pseudo-rotation that could result in an additional offset. For each experiment, the baseline correction was performed using pure water and a preset operating condition. Each concentration was measured for 15 min, and the optical rotation value was averaged across the interval. Different experiments were carried out to evaluate the effect of certain temperatures (298, 313, and 318 K) and variable sampling flow rates (50, 60, and 70 mL/min). The data shown in Figure 5 show good linearity for all flow rates and temperatures and further show that the temperature has only a minor effect. Changing the flow rate also seemed not to affect the measurements negatively.

### 2.4 Overview of the Automated Sampling Process

**Scheme 1:**
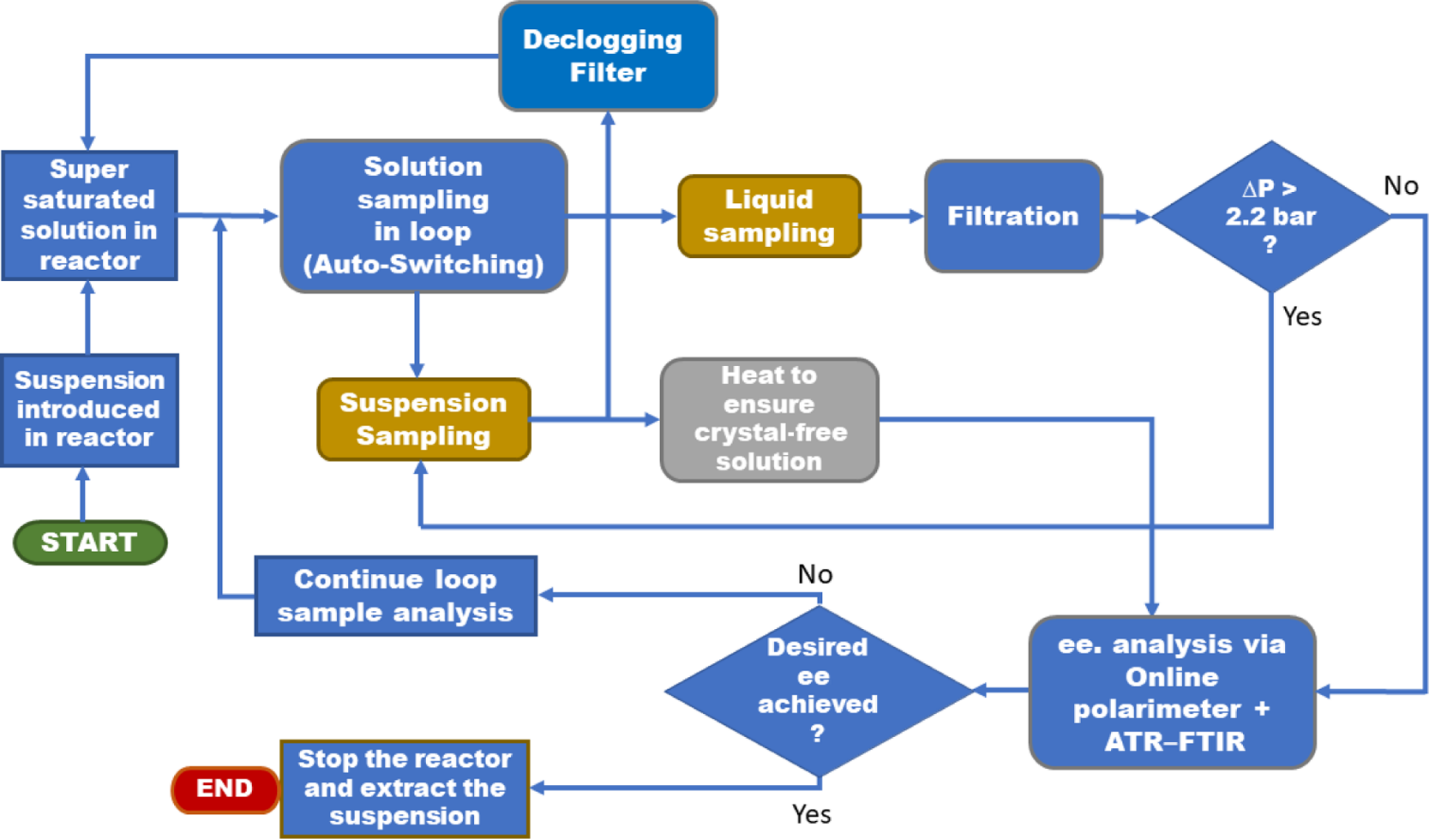
Block Flow Diagram of the Automated Sampling Loop Setup for Chiral Crystallization Process Monitoring Starting with saturated solution sampling in the loop and optimizing *T* and flow rate. Liquid/suspension sampling was performed by autoswitch mechanism, and *ee* was analyzed using a polarimeter and an ATR–FTIR system.

Scheme 1 shows an overview of the automated sampling of the crystallization process. It describes the steps that the sample suspension goes through during the whole loop cycle. Starting from the sample solution being pumped in the loop and acquiring steady operating condition required for ensuring accurate analysis. Suspension is then introduced into the reactor and then pumped into the loop for liquid and suspension sampling. Automated switching between both modes of operations was enabled with the help of pressure sensors (across the filter). Crystal-free heated solution is pumped into the analytical loop section, where enantiomeric excess is measured using the ATR–FTIR probe and in-line polarimeter. The sample is analyzed in a continuous loop until the desired enantiomeric excess is achieved. This enables the process to be stopped at the optimum time and to obtain the desired crystal product from the solution.

## 3 Process Modeling

The process modeling work focused on analyzing the operating limits of the system in terms of temperatures and flow rates as well as the underlying kinetics of dissolution. As the setup aims to dissolve crystals in the loop at higher temperature, it is critical to identify the working temperature range of the vessel and the sampling setup as well as to rationalize the suspension densities and particle sizes that can be tolerated. The model assumes a steady-state condition, and for simplification, the following assumptions are applied: (a) the jacket temperature is uniform with heat transfer only occurring from the crystallizer to the jacket (i.e., the jacket is perfectly insulated to the outside) and b) the energy released by crystal nucleation and growth is neglected. The process is modeled using the energy balance equations for the co-/counter-current heated tube segments and the crystallizer using the PBEs and the MBEs.

To evaluate the operating limits in terms of suspension density (maximum limited by the dissolution rate and temperature), there is a need to analyze the evolution of crystals along the sampling loop using a simplified population balance model that was derived by Iggland and Mazzotti.^49^ Dissolution variability is accounted for with different activation energies.^48^

This section consists of two parts: the first describes the energy balance equations for the crystallizer vessel and the sampling loop, followed by a section describing the PBEs. The derivation of the model equations is explained in Section S2. For determining the suspension dissolution (operating limit) in the loop, the effect of the dissolution rate on PSD is critical, for which a simple PBE model is developed for the heated loop.

### 3.1 Energy Balance for the Crystallizer and Sampling Loop

The energy balance equations are derived in order to predict the required jacket thermostat temperature *T*_j_ to achieve the target vessel temperature *T*_v_ due to the heated solution coming into the vessel at *T*_loop_. The analysis can be conveniently divided into three sections: (1) determination of the vessel heat transfer coefficient *U*_v_ with no flow in the loop; (2) determination of the steady-state vessel temperature with an active loop; and (3) energy balance equation for loop segments.

#### 3.1.1 Determination of the Vessel Heat Transfer Coefficient *U*_v_ with No Flow in the Loop

The heat transfer coefficient of the vessel, *U*_v_, can be determined from the integration of the energy balance equation for the crystallizer with no flow in and out of the vessel over a time period from *t* = 0 to *t*. At time *t*, the temperature in the crystallizer, *T*_v(*t*),_ is given by
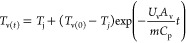
4where *T*_v(0)_ is the temperature at *t* = 0 (eq S7 in the Supporting Information). This equation was used to determine *U*_v_ via a curve fitting method.

#### 3.1.2 Determination of the Steady-State Crystallizer Vessel Temperature with an Active Loop

In order to determine the effect of the heated loop solution (*T*_loop_) coming back into the vessel at a volumetric flow rate (*Q*), we further develop the energy balance equation to identify the preprocess temperature range in the loop and target crystallizer temperature. The energy balance equation can be written as (after eq S8)
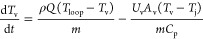
5

Considering this equation, we have the following variables *Q*, *T*_loop_, *T*_v_, and *T*_j_ and constants *m*, *C*_p_, *U*_v_, and *A*. Assuming steady state, that is, , and with similar assumptions to those made for eq 4, this gives us the following equation for the temperature of the crystallizer vessel
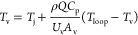
6

Equation 6 provides us with threshold temperature ranges of the vessel and loop that can be achieved at a certain flow rate. Similarly, for the case of suspension sampling and back-purge, a two-stream flow is considered split in equal halves (accomplished by a control valve). The modified model equation for the two-stream mode while considering the steady-state condition and assumptions can be written as follows
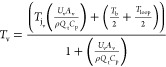
7

These two model equation, eqs 6 and 7, can be solved together to determine the overall heat transfer across the loop and the loop temperature limitations at a given flow rate.

#### 3.1.3 Energy Balance Equation for Loop Segments

**Figure 6:**
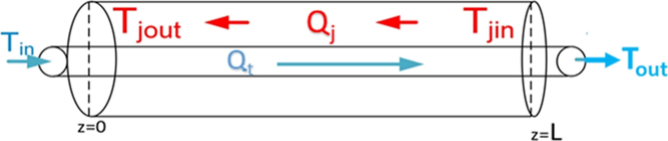
Tube-in-tube loop segment: Counter-current flow is occurring between the two streams where the sampling tube inlet temperature changes from *T*_in_ (cold) to *T*_out_ (warm) at a volumetric flow rate *Q*_t_ and jacket temperature *T*_jin_ hot to *T*_jout_ cooler at a flow rate *Q*_j_.

The sampling loop consists of multiple heating tubes in segments to ensure that undersaturated conditions are maintained inside the loop segment, as shown in Figure 3. The complete setup consists of five sampling loop segments and a crystallizer vessel segment. For this, we consider a cross-section of a tube in a heat exchanger segment with length *L* shown in Figure 6. Assuming steady-state conditions, the energy balance equation for the sampling tube and jacket tube can be derived as follows
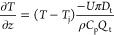
8where *D*_t_ is the diameter of the tube. For the jacket tube, the equation can be written as
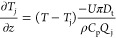
9

Assuming that *U* on both sides of the tube is the same, then combining both eqs 8 and 9 and integrating with limits gives the energy balance of the whole segment
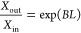
10where *X* = *T* – *T*_j_ and *B* = . For each segment, two equations are derived to solve the temperature across that segment. For example, for loop segment 1, equations are written as follows
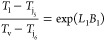
11
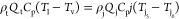
12

These two are the energy balances over the tube-in-tube heat exchangers. Equation 11 states that energy (heat) gain between sample tube ends should be equal to energy (heat) loss between jacket tube ends. Equation 12 defines the energy balance across the segments.

Similarly, all the equations for the other segments can be derived. Considering the flow sequence of each mode of operation, this gives us a set of 11 equations for each mode of operation and requires us to solve 11 unknowns to determine possible model solutions for the temperature profile in both sampling mode sequences that includes all five loop tube segments and the crystallizer vessel.

### 3.2 Population Balance Equations

For determining the suspension dissolution along the loop, the effect of dissolution rate on the PSD is developed for the sampling loop. The PBE of the heating loop will behave in a similar way to a plug flow reactor, but in our case, only growth or dissolution is assumed and nucleation, attrition, and agglomeration are neglected for the purpose of modeling. The PBE can be then written as
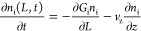
13where  represents the PSD,  represents the growth and dissolution term, and  represents the flow term with *v*_z_ as the flow velocity. Assuming a steady-state condition, . (i = R and S enantiomers)
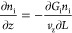
14

The population balance is linked with the MBE to track concentration changes in the solution phase. The MBE for two enantiomers is (i = R and S enantiomers)
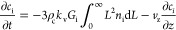
15where ρ_c_ is the density of crystals and *k*_v_ is the shape factor with values used listed in Table 1. A spherical shape factor is assumed for simplicity of modeling, although the observed needle-like crystals were ground into fines with the aid of the dispersing tool. Applying steady-state conditions to this equation , MBEs can be written as (i = R and S)
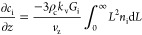
16

**Table 1:** Model Parameters Values^a^

parameter	notation	value
activation energy of dissolution	*E*_d_ [kJ kmol^–1^]	12,000
pre-exponential factor of dissolution	*k*_d_ [μm s^–1^]	500
universal gas constant	*R* [kJ kmol^–1^ K^–1^]	8.314
heat capacity	*C*_p_ [J kg^–1^ K^–1^]	4186
diameter of loop segments 1 and 5	*D*_t1,t5_ [m]	6.35 × 10^–3^
diameter of loop segments 2 and 3	*D*_t2,t3_ [m]	3.175 × 10^–3^
diameter of loop segments 4	*D*_t4_ [m]	5.64 × 10^–3^
heat transfer coefficient of loop segments 1	*U*_1_ [J m^–2^ K^–1^_S_^–1^]	64
heat transfer coefficient of loop segments 2 and 3	*U*_2_ [J m^–2^ K^–1^_S_^–1^]	121
heat transfer coefficient of the measuring cell	*U*_4_ [J m^–2^ K^–1^_S_^–1^]	350
heat transfer coefficient of the crystallizer	*U*_v_ [J m^–2^ K^–1^_S_^–1^]	86
density of water	ρ [kg m^–3^]	997
mean particle size	λ_1,i_ [μm]	50
solubility parameter *q*_0_	*q*_0_ [g g^–1^]	0.64
solubility parameter *q*_1_	*q*_1_ [K]	273
l-asparagine M crystal density	ρ_c_ [kg m^–3^]	1300
crystal shape factor	*k*_v_	π/6
initial sample temperature	*T*_in_ [K]	293
jacket temperature for the heating tube	*T*_jin_ [K]	336
sampled solution flow rate	*v*_z_ [mL min^–1^]	50
jacket solution flow rate of water	*Q*_j_ [m^3^ s^–1^]	22
total sample tube length	*Z* [m]	1.75

a Including physiochemical parameters and constants that are applied in the model equation simulations.

**Table 2:** Table of Notations Used^a^

name	symbol	unit
specific optical rotation	α_λ_^T^	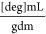
optical rotation	α	[deg]
path length measuring cell	*l*	dm
loop residence time	τ_loop_	min
loop volume	*V*_loop_	m^3^
vessel temperature	*T*_v_	°C
temperature sampling tube	*T*_i_	°C
tube jacket temperature	*T*_ji_	°C
vessel jacket temperature	*T*_jv_	°C
loop temperature end of the loop	*T*_loop_	°C
back-purge stream temperature	*T*_b_	°C
length of tube segments	*L*_i_	m
diameter of the tube	*D*_ti_	m
radius of the tube	*r*_t_	m
mass of solution/sample	*m*	kg
density of water	ρ	kg/m^3^
area of the vessel	*A*	m^2^
surface area of the vessel	*A*_v_	m^2^
mass flow rate	m*˙*	kg/s
volumetric flow rate of the tube segment	*Q*_ti_	m^3^/s
volumetric flow rate of the jacket fluid	*Q*_ji_	m^3^/s
overall heat transfer coefficient of the loop	*U*	W/K m^2^
heat transfer coefficient of the vessel	*U*_v_	W/K m^2^
heat capacity	*C*_p_	J/kg K
flow velocity	*v*	m/s

a Table 2 enlists the notations used in the equations above.

The initial and boundary conditions for PBEs and MBEs are written as follows
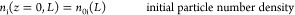
17
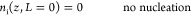
18
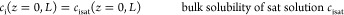
19where i = R and S. Second- or higher-order spatial accuracy is obtained in the smooth parts of the solution, and first-order spatial accuracy is obtained in regions with large gradients.^55^ The scheme is shock-capturing and preserves monotonicity, thereby ensuring stability of the numerical method.

## 4 Results and Discussion

### 4.1 Overview of Experiments

The experimental study was based on demonstrating a working proof of the sampling setup and its operating modes, that is, the liquid and suspension sampling modes, using l-asparagine (LAM) as a model compound. Initial experiments were attempted to investigate the jacketed vessel and loop steady temperature at variable flow rates. How different modes of operation affected the temperature profile across the loop to attain the desired target temperatures at the measuring cell was also observed. Prior to the main experiments, this step was critical for checking the process parameters. The temperature and flow rate steady set point were crucial for acquiring accurate sampling and polarimeter baseline settings, and calibration experiments of the analytical equipment were evaluated by using suitable sampling flow rates and vessel and loop temperatures. In the cyclic mode test, we show that the automated sampling and declogging of the filter system results in a stable operation of the analytical device. In the later suspension sampling mode, steady-state experiments were operated to assess the consistency of the measured data, and the dispersing tool was applied to observe its effect on the sampling analytical data. For all these experiments, the known weight % of crystal suspension for the optical rotation analysis was used. As the total concentration for these experiments was known and remained constant, ATR–FTIR analysis was not required. Calibration results of the ATR–FTIR sampling loop experiments performed at variable temperatures are shown separately in the Supporting Information.

For the sampling mode experiments, critical parameters such as loop flow rate *Q*_loop_, dispersing tool speed, loop residence time (τ_loop_), dissolution rate, and pressure drop during filtration and declogging were investigated. This experimental proof of concept is combined with a modeling study elucidating the operating range of the measurement device in terms of temperatures, flow rates, and the underlying kinetics of dissolution (and liquid-phase racemization in the case of VR).

The process was modeled using the energy balance equations for the co-/counter-current heated tube segments and the crystallizer, PBEs and MBEs, as described in the previous section Section 3.

### 4.2 Operating Limits of the Setup

**Figure 7:**
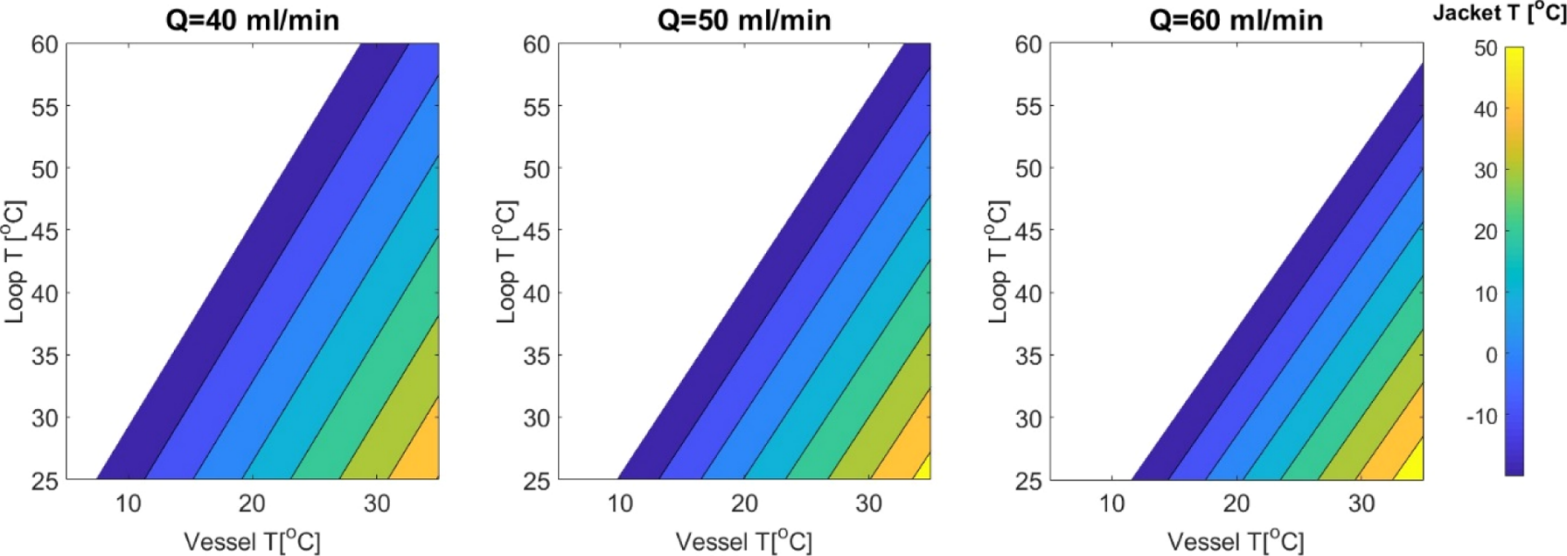
Liquid sampling: numerical solution of the operating limit working *T* range for the vessel *T*_v_ and loop temperature *T*_loop_ at a variable flow rate. The color bar indicates the jacket *T* range. It is required to achieve the desired vessel *T* at a specific flow rate of the loop stream at constant loop *T*. Each graph displays the workable regime at a certain flow rate for varied loop *T*.

To determine the temperature range design space, the overall heat transfer coefficient *U*_v_ of the crystallizer must be determined. For this purpose, tests were carried out to record the temperature profile by cooling the vessel at constant rate and maintaining no flow in and out of the vessel. Experimental data were then compared with the numerically calculated data using eq 6, and curve fitting (lsqcurvefit method in Matlab) was applied. The value of the heat transfer coefficient determined by this method showed a very good fit with the experimental results, and the *U*_v_ obtained was 86 J/(m^2^ K s). The assumption made in the equation was that there was no flow in and out of the vessel. As our setup aims to dissolve crystals in the loop at higher temperature for accurate measurement, it was crucial to identify the working temperature range of the vessel and sampling setup. Model eq 8 was used to identify the operating limits due to the effect of the heated solution flowing into the vessel at a flow rate *Q*. It was used for predicting the required jacket thermostat temperature *T*_j_ to achieve the target vessel temperature *T*_v_ due to the heated solution *T*_loop_ coming back into the vessel. Figure 7 shows the numerical solution for the liquid sampling operating mode. The color bar and contour indicate the vessel jacket temperature *T*_j_ ranging from −20 to 60 °C. The lower blue region was restricted to −20 °C due to the limitations of the vessel Thermostat 1. Based on this, each graph shows the working temperature region at a specific flow rate. Shifting from a lower flow rate of 40 mL/min to a higher flow rate of 60 mL/min reduces the operating region since it is limited by jacket temperature restrictions and it requires more vessel cooling power to maintain a steady-state condition. Depending on the solubility data of the model compound shown in Table 1, *T*_j,_*T*_loop_, *T*_v_, and flow rate can be acquired by screening through this predicted operating regime.

**Figure 8:**
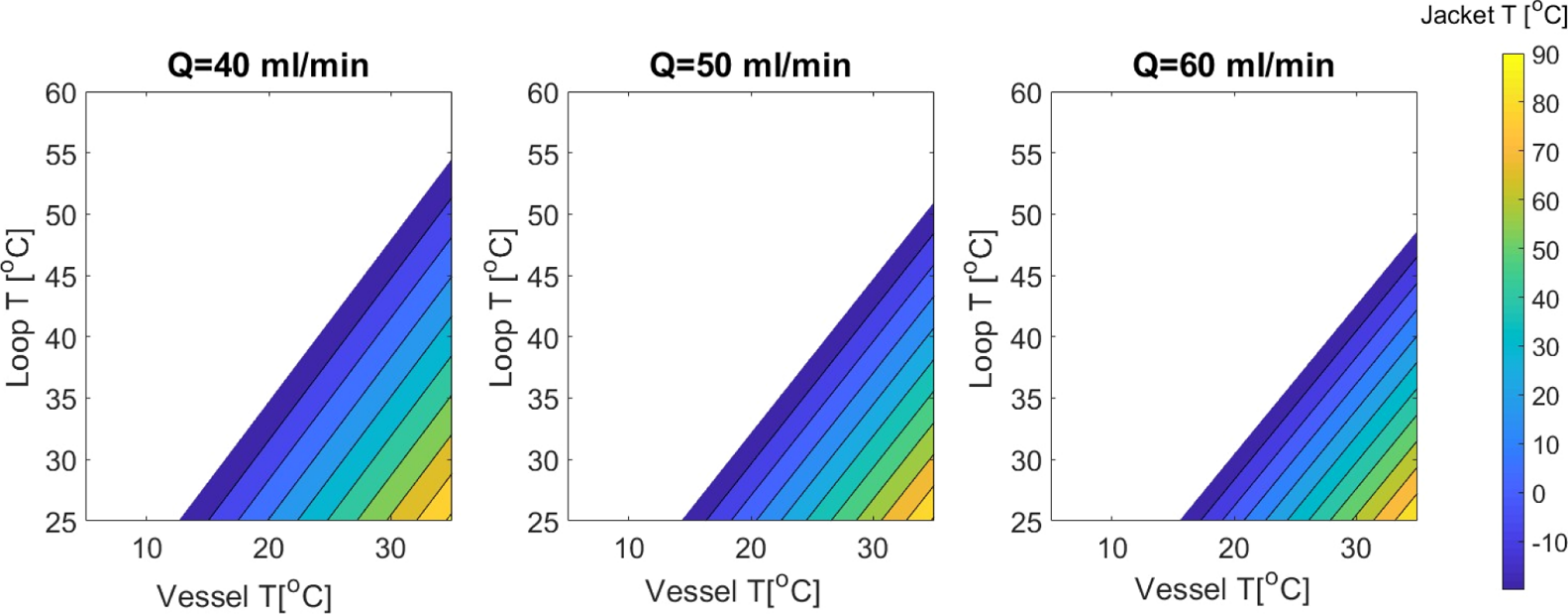
Suspension sampling: Operating limits (numerical solution of double incoming loop streams (suspension sampling and back-purge) affecting the overall temperature range for crystallizer *T*_v_ at a variable flow rate.) This shows that with two streams, the working region shrinks further due to restricted jacket *T* cooling power.

Suspension sampling includes the back-purge stream *T*_b_ and *T*_loop_ flowing back into the vessel at a certain flow rate *Q*. To govern its effect on the vessel temperature region *T*_v_, the model in eq 9 was based on similar assumptions and considered the stream flow as split in an equal ratio. Figure 8 shows that as compared to a single heated stream going back to the vessel, double heated streams reduce the vessel temperature region further, and consequently, there is a more limited process operating range. As in our case, we have to apply both modes of operation alternately, so both working temperature regions must be linked together. Obviously, in this case, the design space is reduced further because of thermostat restrictions. The actual goal of the setup will be able to carry out a crystallization process by maintaining the set lower temperature of the vessel. Therefore, more energy would have to be removed, and the actual operating region of the setup is likely to be smaller.

### 4.3 Temperature Distribution in the Loop

To determine a possible model solution for the sampling setup, the overall energy balance for the heat exchanger segments must be solved for both modes of operation. Eleven sets of equations with 11 unknowns were numerically solved using the optimization toolbox in Matlab (fmincon and fsolve). Heat transfer coefficient values were determined using these experimental temperature sensor data integrated with the energy balance model equations. Jacket *T* (*T*_jv_ = −10 °C and *T*_jloop_ = 50 °C) was fixed and the flow rate was varied for comparison.

**Figure 9:**
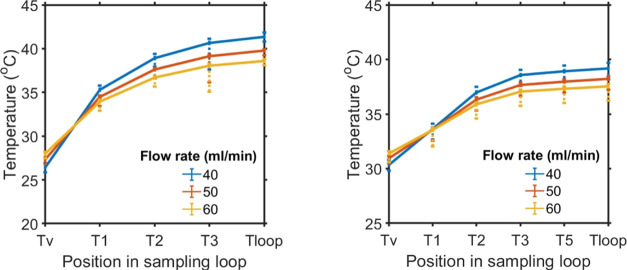
Model solution for temperature points in the loop by keeping the vessel and loop jacket temperature fixed: (left) Temperature distribution during liquid sampling and (right) temperature points in the loop due to the effect of two incoming loop streams during the suspension and back-purge mode. The error bars display the experimental data obtained from temperature sensors using similar heat transfer coefficients to those listed in Table 1, showing a negative offset of 1–2 °C (five replicates for each flow rate).

Figure 9 shows the numerical solution for the temperature profile along the positions in the sampling loop during both modes of operation. The loop positions are indicated in the schematic in Figure 3. Results show that while keeping the jacket temperature constant for both liquid and suspension modes, an increase in flow rate increases the vessel temperature but decreases the temperature across the points in the heated loop. If both modes of operations are compared, the vessel temperature during suspension sampling jumps around 3–4 °C more than that in the liquid sampling, and this is because the double stream (including the back-purge) causes the temperature of the vessel to rise with the similar cooling condition. This means that to maintain the vessel temperature, more cooling power of the vessel thermostat will be required. These numerical results were tested and compared with temperature data obtained from experiments. The trend can be compared with the experimental temperature values; however, there was a small offset of 1–2 °C from the actual experimental data, which can be neglected due to heat loss occurring between loop segments. As these model solutions were assumed to be an ideal case where no heat loss with the surroundings was considered, jacket temperature derived with this model was consistent throughout the loop.

### 4.4 Model Solution: Crystal Size Distribution along the Loop

For determining the suspension dissolution (operating limit) in the loop, the effect of the dissolution rate on PSD is critical, for which a PBE and MBE model was developed. The PBE model for the loop described the evolution of the PSDs, whereas the solution-phase MBE tracked concentrations of the enantiomer under steady-state conditions. The temperature profile along the positions/segments in the sampling loop during both modes of operation were applied to determine its effect on the crystal size distribution. The initial size distribution was based on the initial mean crystal size obtained experimentally. Initially, the crystal suspension is considered suspended in saturated solution.

**Figure 10:**
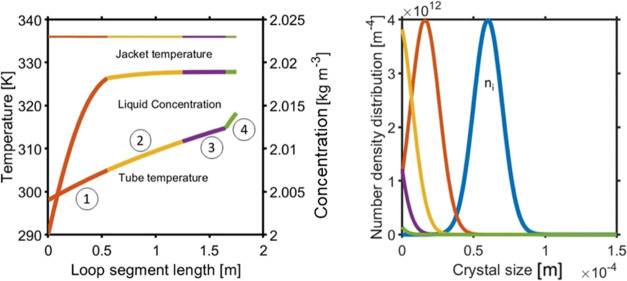
Model solution of PSD for temperature points in loop segments 1, 2, 3, and 4 by keeping the vessel and loop jacket temperature fixed: (left) sample tube *T*, jacket *T*, and liquid phase concentration evolution (right) PSD at the outlet of each loop segment. Initial PSD and distribution along the end of each segment 1, 2, 3, and 4 (colur contrast).

**Figure 11:**
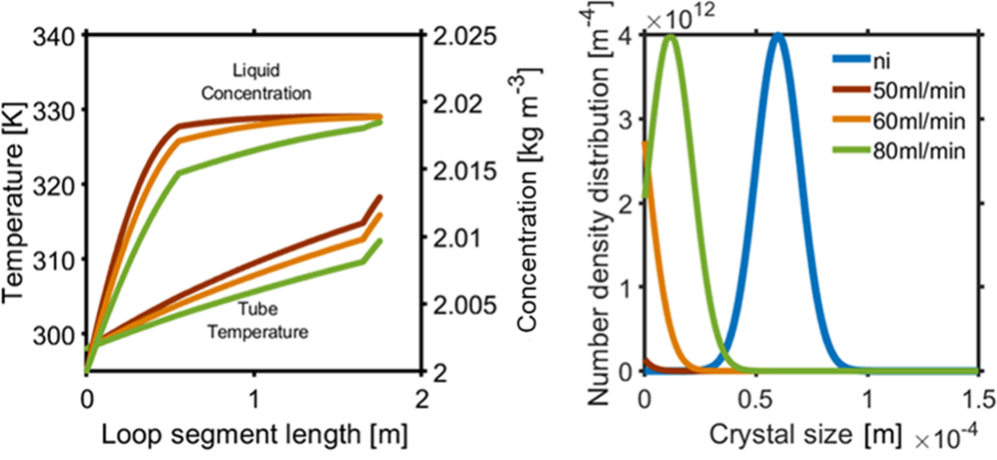
Model solution of PSD for a variable sampling solution flow rate across the loop segment length. Its effect on (left) sample tube temperature, and liquid phase concentration, as well as (right) initial PSD (ni) and final PSD at the end of each loop segment.

Figure 10 shows the effect on the initial crystal PSD by combining the effect of the temperature distribution of the loop segment by solving the PBE and MBE models simultaneously along the loop segment axis. Figure 10 (right) shows the PSD at the outlet of each segment. The PSD at the outlet of each segment indicates that particles are dissolving along the loop segments 1, 2, 3, and 4, as shown by the color contrast. Starting loop segment 1 at higher temperature from the saturated solution temperature, particles dissolve along the segments 1, 2, 3, and 4, which results in an increase in liquid phase concentration. As it reaches the end of segment 3, almost all the crystals are dissolved as there is no longer an increase in liquid concentration, as determined by the MBE. Using this model, we can get an idea as to whether a certain suspension will be completely dissolved in the specific loop temperature range before it enters the analytical segment region, that is, the ATR–FTIR tank and polarimeter measuring cell. The model was further tested by investigating three different sampling flow rates. All other parameters including the initial PSD were kept the same. Results in Figure 11 show the effect of flow rate variation on sampling tube temperature. As the dissolution rate is temperature-dependent, it gives rise to an increase in liquid phase concentration. Table 1 gives the dissolution rate including the other kinetic constants and parameters utilized in these simulations. Figure 11 (right) displays the final PSD obtained at the end of the loop. At a flow rate of 50 mL/min, the final PSD displays a flat line at the end of the loop, which indicates complete dissolution of particles. However, for the same initial distribution, when the flow rate is increased, some of the particles remain undissolved. If the flow rate is increased further to 80 mL/min, the final PSD shifts toward the left but is still completely visible. This indicates that particle size has been reduced but still exists in the sample as a suspension. This is further verified by the liquid concentration MBE evolution, in which at low flow rates, it quickly reaches its maximum concentration in the liquid phase, while at higher flow rates, it is increasing more slowly and requires a longer segment to reach a constant value. Overall, this analysis shows us how flow rate affects the tube temperature profile and which flow rate range can be used for the loop in order to ensure that all crystals are dissolved for a known crystal suspension.

**Figure 12:**
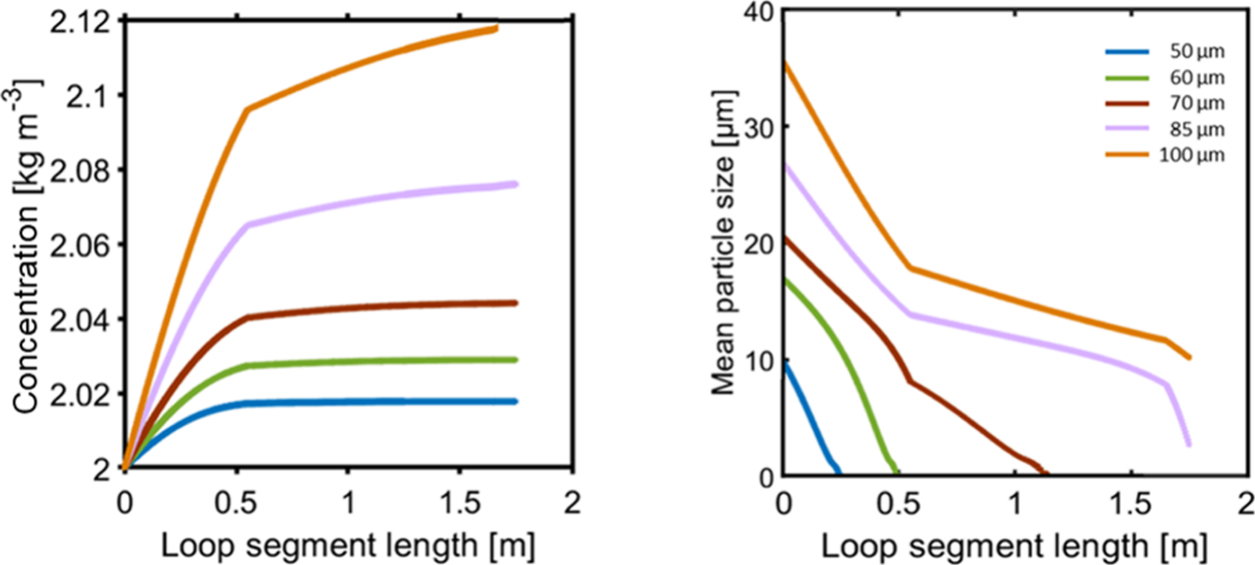
Model solutions of the loop segment model, varying the mean crystal size and keeping the flow rate and vessel and loop jacket temperatures fixed: (left) liquid phase concentration and (right) mean crystal size progression in the solid phase across the segment length.

Figure 12 displays the simulation run of the sampling loop model for different mean particle sizes of the initial PSD. The selected initial distribution is a standard Gaussian distribution, and the mean size denotes the peak of each distribution to be investigated. All remaining parameters are fixed to observe the effect of mean particle size on the liquid phase concentration and particle size across the loop segment. The average length (volume weighted) was calculated by ratio of the fourth and third moment equation written below.
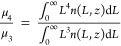
20

Results show that if the distribution of a small mean particle size is chosen (50–60 μm), particles are depleted at less than half of the loop segment length, which means that size sharply reduces to 0 μm. Concentration remains constant after a 0.5 m segment length, indicating there are no longer crystals present in the solid phase. When mean size is further increased, it reduces slowly and requires longer segment lengths to completely dissolve the crystals. Increasing the mean size further leads to a point where it is far away from fully dissolved and the concentration keeps increasing until the end of the segment length. The shift in between the lines is due to the effect of different heat transfer coefficients shown in Table 1 and tube diameters across different loop segments. These results help us evaluate for a given suspension density what mean particle size range is suitable for the defined dissolution rate and temperature. Experimentally, retaining the mean particle size in this range can be achieved by using the correct dispersing tool parameter. Other solutions can be to increase the tube temperature further to dissolve crystals with higher initial mean size distributions.

### 4.5 Filter Purge Performance for Liquid Sampling

**Figure 13:**
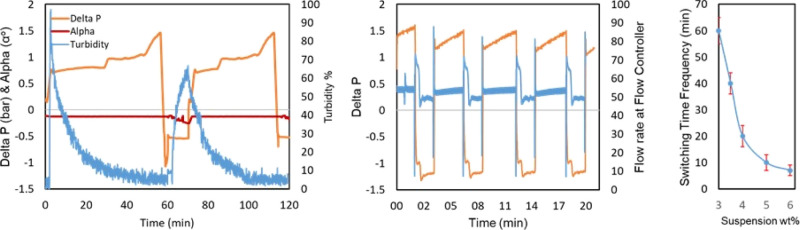
Cyclic liquid sampling experiment: (left) 3 wt % L-AM in saturated solution. Optical rotation (showing the saturated solution rotation angle) and turbidity % (exponential decay of suspension in the vessel during sampling). Delta *P* is the pressure drop measured across the filter (middle). 6.5 wt % suspension auto-switch profile (right). Sampling time switching frequency with respect to varied suspension wt % (error bar indicates at least three repetitions of experiments).

Various experiments were performed to test the filtration performance for liquid sampling. For all tests, the vessel was kept at 25 °C for the crystallizer solution and the temperature of the end segment of the loop reached 45 °C while maintaining undersaturated conditions. Sampling of the suspension was performed at 50 mL/min, and the cyclic sampling mode results are shown in Figure 13. Liquid sampling experiments were performed by varying the suspension density (3, 3.5, 4, 5, 6, and 6.5 wt %) of LAM. In the left panel of Figure 13, the experiment with 3 wt % suspension density is shown. The turbidity probe located in the crystallizer detects an exponential decay of mean particle size in suspension. The optical rotation of the filtered liquid remained constant for all experiments due to the same saturated batch solution. During the filtration mode, the pressure drop slowly increased to 1 bar in around 30 min, which is the result of crystals being stacked along the filter mesh. The gradual accumulation of solids on the filter surface causes an increase in the hydraulic resistance of the filter to the fluid, which results in an increased pressure drop and eventually results in a sharp increase to 1.5 bar, resulting in activation of the timed suspension sampling (preset pressure drop across the filter system was reached). In this, the sampled suspension is pumped through the heated tube segments, and the flow splits into two streams (ensured by the resistance via a flow controller) where one goes toward the analytical equipment and the other is back-purged through the filter segments. This mode runs for a short interval of back-purging, indicated by a negative pressure drop, which allows clogged crystals to stream back into the vessel that results in an increase in turbidity. This cyclic sampling can be repeated frequently until the end of the crystallization process. The threshold pressure limit for switching was set to prevent the filter becoming fully blocked before the back-purge mode is activated. The middle panel in Figure 13 shows a similar type of experiment with a higher suspension density of 6.5 wt %, resulting in a shorter switch time between liquid sampling and the back-purging mode. The dependence of the switching time (minutes) on suspension density is shown in the Figure 13 panel on the right. The switching time frequency was dependent on how quickly the sampled suspension reaches the set threshold pressure drop limit. For the 6 wt % test, the pressure drop starts building up sharply from 1.5 bar and within a few minutes jumps close to 7 bar (to test the pressure relief valve set limit) resulting in a halt in the solution flow and indicating that the filter is fully clogged. Thus, to prevent complete clogging of the filter, the maximum mean set pressure drop threshold was set to be 1.5 bar. The remaining test results for 6, 4, and 3.5 wt % L-AM suspension require switch times of around 8, 20, and 40 min, respectively. This affirms that with the increase in suspension wt %, the filter gets clogged more quickly, requiring more frequent back-purging to declog the filter.

### 4.6 Steady-State Experiments and Use of the Dispersing Tool

**Figure 14:**
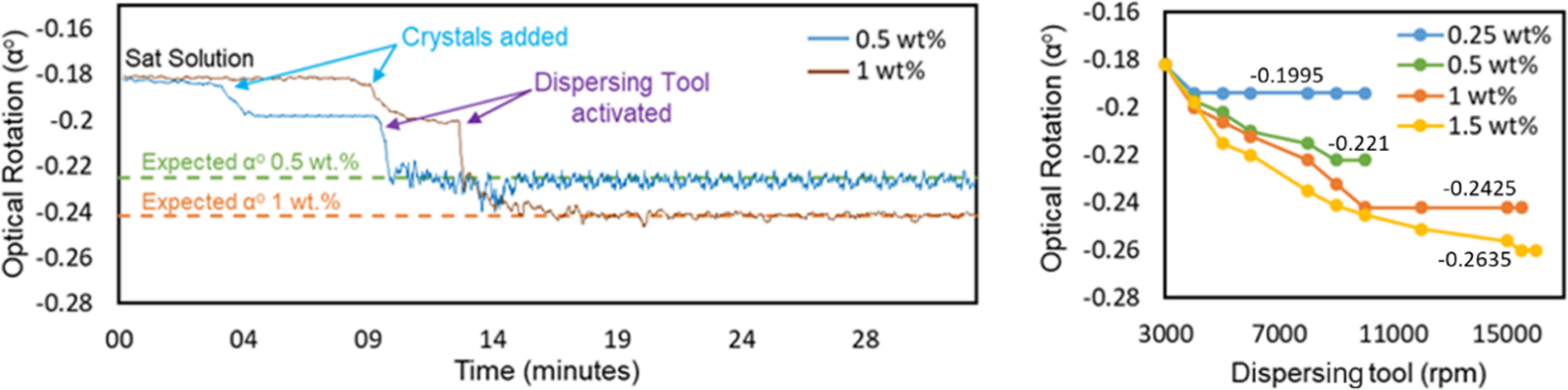
Steady-state continuous suspension sampling: (left) effect of the dispersing tool for accurate optical rotation measurement (moving average method was applied to minimize noise in data). Steady-state continuous LAM suspension sampling (right). Dispersing tool settings (rpm) for attaining the expected rotation angle value.

It is well-known that transporting particles from a stirred vessel into a pipe (e.g., into the sampling loop) can lead to significant size classification issues, especially for broad PSDs. To investigate this for the case of our setup, we performed a series of steady-state experiments. The experiments were initiated by introducing a saturated solution into the vessel, controlled at 25 °C, while the sampling solution was heated to 45 °C in the loop to ensure that crystals were dissolved before entering the measuring cell. Experiments were performed by varying the flow rate (50–80 mL/min) and suspension densities (0.25–2 wt % L-AM crystals). In these initial suspension sampling experiments, rotation angles consistent with the calibration data in Figure 5 were never obtained. A closer inspection of the vessel content and the particles entering into the sampling loop revealed that bigger crystals could not enter the sampling loop, thus leading to a biased sample and erroneous optical rotation angle measurements. Both enantiomers grow at the same rate, and thus, large crystals are not sampled in an appropriate proportion that is representative of the suspension inside the crystallizer. This can lead to inconsistency in the polarimeter results. Since the maximum flow rate that can be used in the sampling loop is inherently limited by the dissolution kinetics, as well as the performance of the thermostats, another solution had to be devised. To this end, a dispersing tool was installed. Results of the steady-state experiment investigated for 0.5 and 1 wt % suspensions using the dispersing tool are shown in Figure 14 (left). An L-AM saturated solution was introduced after reaching the stable operating settings and baseline, with weighed crystals introduced into the crystallizer. The suspension was sampled, and within a few seconds, a shift was observed in optical rotation. The measured alpha value (−0.195°) was not close to the anticipated value. This was observed for around a 10 min duration. Afterward, the dispersion tool was activated, resulting in the milling of crystals into fines, and a change was observed immediately in the alpha value, reaching the accurate 0.5 wt % alpha value (fit with the calibration curve from Figure 5). Other experiments evaluated with 1 wt % suspension and similar process conditions were applied, showing that when crystals were added in the vessel, the alpha value shifted but was considerably different from the expected value (−0.199°). Applying the dispersing tool resulted in an accurate (−0.241°) alpha value. However, for higher suspension densities, adjustment of the dispersing tool to 10,000 rpm and the minimum flow rate to 50 mL/min was required to acquire the expected optical rotation value.

Higher density suspensions up to 2 wt % were examined, but it required adjusting parameters such as a higher rotational speed of the dispersing tool and a higher loop temperature to attain accurate results. For stability, all tests were carried out for around 1 h, and the alpha values obtained were consistent and steady. Noise was an issue when using the dispersing tool due to the generation of bubbles (from the high rotational speed of the shaft) in the solution. This was minimized numerically by using a moving average method. However, the higher suspension density experiment showed that larger optical rotation signals were observed with relatively less noise. Thus, for different suspension densities, the dispersing device was adjusted at a higher rotation value, which enables the optical measurements to be made with improved accuracy and without using a numerical smoothing function.

## 5 Conclusions

Chiral resolution processes are used for the manufacture of APIs in their purest form. An integral part of this process is separation and purification, which is carried out by crystallization processes. The quality of the product is determined by the size, shape, form, and purity of the material. Obtaining the desired properties requires accurate design and control of the crystallization process, and one of the main challenges is to control these parameters while optimizing the process. Utilization of the PAT approach provides an opportunity to observe and optimize chiral processes. A polarimeter in combination with an ATR–FTIR probe can prove to be a powerful and reliable combination for the monitoring of chiral resolution processes. The polarimeter is used for measuring the optical rotation angle of active chiral compounds, while ATR–FTIR measures the total concentration and for the detection of impurities and achiral species. The closed loop automated sampling setup allows real-time continuous monitoring of progress in the chiral crystallization process by analyzing both the solid and liquid phases alternatively, which enables the determination of concentration, yield, and purity of enantiomers present in both phases. Calibration tests of both tools were performed using the appropriate numerical modeling method (PLSR and linear fitting). Liquid sampling cyclic experimental results showed that alternative liquid and suspension sampling can be accomplished, but switching between each mode was critically dependent on the suspension density. In steady-state suspension sampling tests, use of the dispersing tool enabled the optical rotation measurement of crystal suspension to produce the expected value. This is rationalized as an effect of size classification at the inlet of the sampling loop, thus making it beneficial when the dispersing tool is used to reduce the size of the particles in the crystallizer and therefore allow representative sampling. Furthermore, this tool can also be used as an aid in the attrition process and can also play an important role in a VR temperature cycling process. However, for increasing suspension density, it will require adjustments in the dispersing tool power and pump flow rate to ensure that the desired fines are pumped into the sampling loop. The dispersing tool will generate more fines, which will cause faster dissolution of enantiomers and as a result faster racemization in the liquid phase. Thus, the deracemization will complete faster.

These experiments show conceptually that our measurement device can successfully continuously sample the suspension, as well as the liquid phase, and allows a stable operation where the filter system is automatically declogged by switching the flow direction. In order to elucidate the operating envelope and expected accuracy of the measurement device, we have carried out simulations of the sampling loop, where we have accounted for the energy balance in the crystallizer and the sampling loop, as well as described the crystallization and dissolution kinetics acting on the particles using PBEs. In the case of VR, we have further included the racemization reaction occurring in the liquid phase. As one might expect, weakly temperature-dependent compound solubility and heat transfer limitations occurring in the sampling loop limit the operating range of the measurement device in the suspension sampling mode (where it is essential to dissolve all sampled particles before reaching the polarimeter). Furthermore, the faster the racemization reaction in the liquid phase is, the less precise the measurement of the solid-state enantiomeric excess becomes when performing VR simulations. These limitations notwithstanding, we hope that our automated measurement setup will enable us to monitor and eventually control chiral crystallization processes continuously and robustly. The further development of reliable analytical techniques is essential for monitoring the separation progress directly and thus facilitating process control.

A control box GUI was developed using LabVIEW for real-time data plotting. It provides a user-friendly interface for monitoring and control of valve operation modes, sample flow rate, and temperature set point values and displays and records real-time measurement of optical rotation, temperature gradient in the loop, and pressure drop across the filter. In future, we aim to modify this program to automate the process control by acquiring and analyzing data acquired for measurement tools.

In future studies, we plan to integrate the sampling setup with a larger reactor, where in addition, an FBRM probe and a dispersing tool can also be integrated together. This will allow us to control the dispersing tool and monitor the PSD required for efficient sampling and also prevention of agglomeration of crystals. Monitoring of the PC and VR temperature cyclic processes will be executed. The procedure developed in this study combines online PATs with chemometrics statistical tools. This can provide a basis for tackling problems faced during chiral crystallization and opens the door to improved control of the process in a more effective and reliable way.

## Supporting Information Available

Complete derivation of the energy balance model equation for the crystallizer and loop segments, including the vessel and tube-in-tube and loop segments setup schematics (PDF)
