# Supplementary material for: Design, Development, and Analysis of an Automated Sampling Loop for Online Monitoring of Chiral Crystallization
Source: Org Process Res Dev. 2022 Feb 15;26(4):1063–77. doi: 10.1021/acs.oprd.1c00320 (PMC9098190; doi:10.1021/acs.oprd.1c00320)
Supplement: Supplementary file 1 — op1c00320_si_001.pdf [file op1c00320_si_001.pdf]

Supporting Information: Design, development and analysis of an automated sampling loop for online monitoring of chiral crystallization

*Ghufran ur Rehman, Thomas Vetter and Philip A. Martin**

Department of Chemical Engineering and Analytical Science, University of Manchester, M13 9PL Manchester, United Kingdom

1 ATR-FTIR probe calibration

The ATR-FTIR spectroscopy calibration procedure to measure total concentration of both enantiomers in liquid phase is described here. L-Asparagine (LAM) of varied concentrations (10,20,30,40,50 mg/g) was dissolved in DI-Water. All are sampled through the loop at a flowrate of 50 ml/min. Full averaged ATR FTIR spectra (average of 128 scans collected every minute interval at spectral resolution of 8 cm^{-1}) obtained are shown in Figure S1, which shows the fingerprint regions of LAM. Water was collected as a background spectrum to eliminate its IR

contribution as much as possible. The spectral region between 1370 and 1461 cm^{-1} was used for calibration as it is least effected by temperature variation and signal drifts (caused by external effects during continuous monitoring). A linear baseline correction enabled the minimization of these effects and resulted in a more robust and accurate concentration estimation.

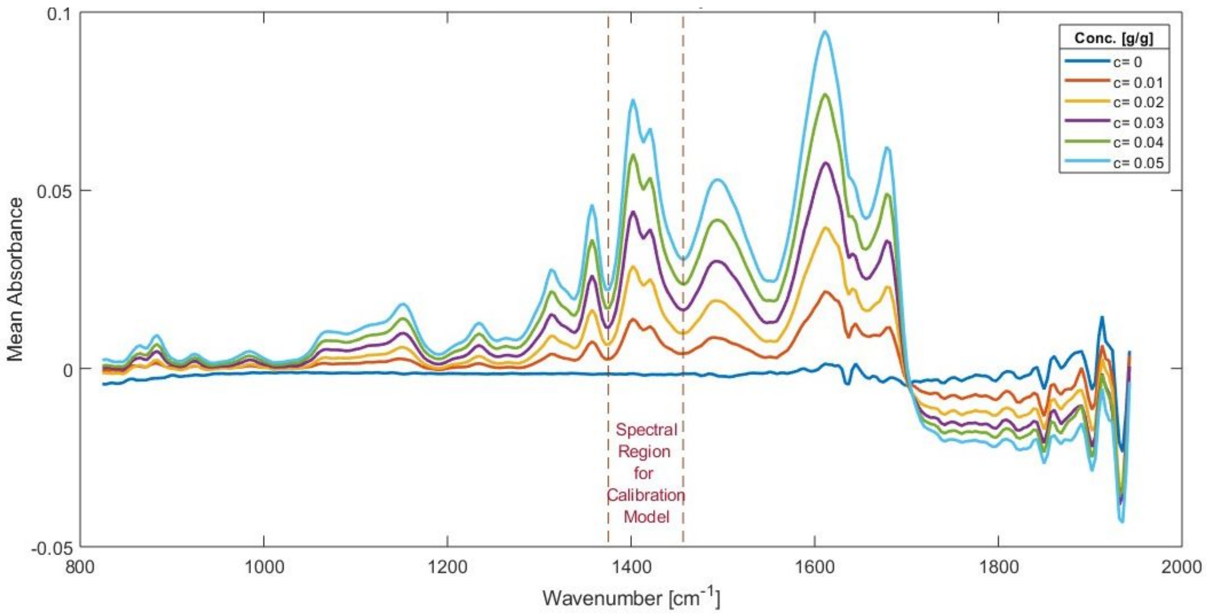

Figure S 1. Full averaged absorbance spectra of L-Asparagine Monohydrate (L-AM) in water (g of L-AM/g of H_20).(Raw data, no baseline correction), dotted line shows spectral region selected for concentration calibration model

As the temperature in the loop can vary during sampling, temperature calibration was also taken into account in the multivariate model. In the model, 25 variables (wavenumbers) were taken in the spectral range and these were tested for 5 different concentrations of LAM, first at a flow rate of 50

ml/min and varied loop temperatures (35, 45 and 55 °C) to determine the effect of temperature variation. 180-250 samples were measured every 1 minute and the baseline corrected. A partial least squares regression (PLSR) model was then used to correlate the spectral features with LAM concentration[1,2]. This is shown in figure S2, where the PLSR model calibration curve with two latent variables (L.Vs) correlates well with experimental data. The following Table 1 shows the ATR-FTIR calibration data by PLSR calibration method using 1,2 components.

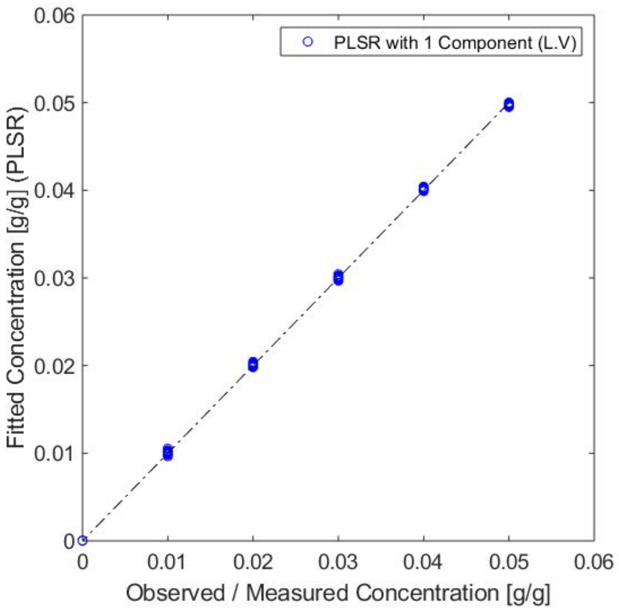

Figure S2. Model Calibration curve: the scatter shows a reasonable correlation between fitted (yfit) and observed (yBlock) concentration responses

Table S1: Partial least squares regression (PLSR) Calibration Method using root mean-squared error of cross validation RMSECV at 50 ml/min flow rate, 25 variables.

Sampling Loop Test Variable Conc.	Method	Spectral region (cm^{-1})	Preprocessing correction	L.Vs	RMSECV (%)	R^2	Variance %
Constant T	PLSR-2C	1370-1461	linear baseline	2	1.6310	0.9998	6.5
Constant T	PLSR-1C	1370-1461	linear baseline	1	1.6310	0.97	6
Varied T	PLSR-2C	1370-1461	linear baseline	2	1.7040	0.9994	5.86
Varied T	PLSR-1C	1370-1461	linear baseline	1	1.7040	0.9918	5.1
Comparison of models	PLSR-2C	1370-1461	linear baseline	2	1.6140	0.9979	6.195
Comparison of models	PLSR-1C	1370-1461	linear baseline	1	1.6142	0.9887	6.195

2 Derivation of energy balance equations

2.1 Crystallizer vessel and thermostat

This section derives the energy balance to determine dT/dt for the crystallizer vessel and thermostat-controlled jacket surrounding it. Consider a jacketed batch reactor shown in Figure S3 with a solution mass m and having a vessel temperature T_v and a thermostat-cooled jacket with temperature T_j, the sample is continuously pumped at flowrate Q_t in a closed loop.

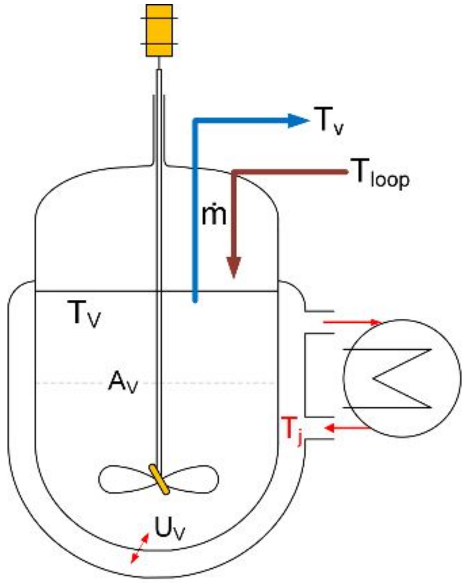

Figure S3. Crystallizer batch vessel of surface area A_v at temperature T_v, with a solution pumped into the vessel from the sampling loop at a specific flow rate $\dot{m}$. A thermostat connected with the vessel keeps the temperature at T_j. T_{in} = T_{loop} (coming from loop) and T_{out} = T_v (going out of vessel).

Considering the ideal batch crystallizer case, the assumptions used are 1) no nucleation and growth occurring, the solution used is pure water and 2) the jacket vessel and loop are perfectly insulated so there is no heat loss/gain across the jacket and vessel.

The energy balance equation states that:

enthalpy of vessel = enthalpy in – enthalpy out + HT (heat transfer)

$$[mC_pT_v]_{t+\Delta t} - [mC_pT_v]_t = \dot{m}C_pT_{in}\Delta t - \dot{m}C_pT_{out}\Delta t - U_vA_v(T_v - T_j)\Delta t \tag{S1}$$

$$\frac{[T_v]_{t+\Delta t} - [T_v]_t}{\Delta t} = \frac{\dot{m}(T_{in} - T_{out})}{m} - \frac{U_vA_v(T_v - T_{out})}{mC_p} \tag{S2}$$

$$\frac{dT_v}{dt} = \frac{\dot{m}(T_{loop} - T_v)}{m} - \frac{U_v A_v (T_v - T_j)}{mC_p} \tag{S3}$$

Table 2 shows a list of notations used in the derivation of the energy balance equation.

Assumption (3) no flow is coming in and out of vessel gives $\dot{m} = 0$ and equation S3 becomes

$$\frac{dT_v}{dt} = -\frac{U_v A_v (T_v - T_j)}{mC_p} \tag{S4}$$

Integrating on both sides with the limits Tstart = $T_{v(0)}$ to $T_{v(t)}$ and $t = 0$ to t gives

$$\int_{T_{v(0)}}^{T_{v(t)}} \frac{1}{(T_v - T_j)} dT_v = \int_0^t -\frac{U_v A_v}{mC_p} dt \tag{S5}$$

$$\frac{(T_{v(t)} - T_j)}{T_{v(0)} - T_j} = \exp\left(-\frac{U_v A_v}{mCp} t\right) \tag{S6}$$

$$T_{v(t)} = T_j + (T_{v(0)} - T_j) \exp\left(-\frac{U_v A_v}{mCp} t\right) \tag{S7}$$

The final equation 7 derived above was used to determine the heat transfer coefficient U between the vessel and the thermostat setup using the Solver method in Excel (sum of errors between calculated and experimental value near minimum approximation = 0 for an exact fit). For the curve fitting, one experiment was performed by cooling the vessel at constant rate and keeping no flow in and out of the vessel. The experimental T_{vessel} vs time obtained was used to estimate the final heat transfer value, which was later applied to the numerical simulations.

2.2 Energy balance equation including vessel temperature and flow rate

In order to determine the effect of the heated loop solution coming back into the vessel at a certain flow rate, we will have to further derive the energy balance equation omitting assumption 3. Equation 3 can be further developed,

$$\frac{dT_v}{dt} = \frac{\dot{m}(T_{loop} - T_v)}{m} - \frac{U_v A_v (T_v - T_j)}{m C_p} \tag{S8}$$

Considering this equation 8, we have the following variables Q, T_{loop}, T_v , T_j and constants m, C_p, U_v and A. We must determine the effect of the heated solution on the jacket temperature T_j required to achieve the target vessel temperature T_v. Assuming steady state i.e. $\frac{dT_v}{dt} = 0$

$$0 = \frac{\dot{m}(T_{loop} - T_v)}{m} - \frac{U_v A_v (T_v - T_j)}{m C_p} \tag{S9}$$

Rearranging equation 9 gives,

$$-\frac{U_v A_v}{C_p} T_j = \dot{m}(T_{loop} - T_v) - \frac{UA}{C_p} T_v \tag{S10}$$

Mass flow can be written as $\dot{m} = \rho$Q and $T_j = T_{jv}$ as the thermostat jacket set-point of loop and crystallizer vessel are separate

$$T_v = T_{j_v} + \frac{\rho Q C_p}{U_v A_v}(T_{loop} - T_v) \tag{S11}$$

Eqn. 11 allows us to calculate the jacket temperature required to achieve the target vessel temperature when a heated solution from the loop is continuously flowing into the reactor vessel. It provides us with threshold temperature ranges of the vessel and loop that can be acquired at a

certain flow rate. This equation 11 was used for acquiring the model solutions shown in section 4.2 of the results and discussion.

2.3 Energy balance equation including loop temperature and back-purge stream temperature at a specific flow rate

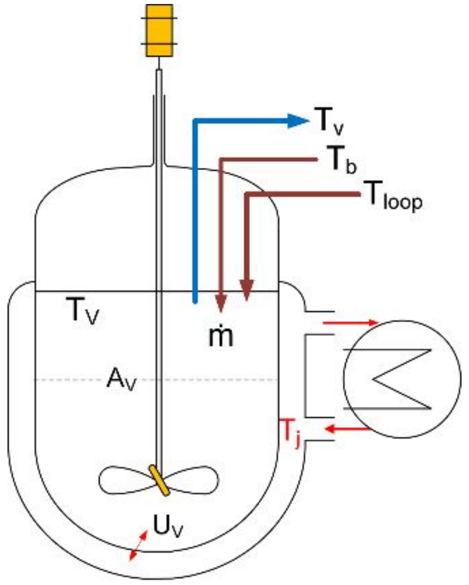

Figure S4. Crystallizer batch vessel of surface area A_v at temperature $T_{v,}$ solution pumped into with sampling loop at a specific flow rate $\dot{m}$ and flowing back into vessel by two heated streams (T_b and T_{loop}).

This section considers the suspension sampling mode when the back-purge returns into the vessel for filter cleaning. For this purpose, consider the outgoing stream from the vessel to be branched equally in two (back purge and end loop stream). The modified energy balance can then be then written as:

Accumulation = In – Out + HT (heat transfer)

$$[mC_pT_v]_{t+\Delta t} - [mC_pT_v]_t = \frac{\dot{m}}{2}C_pT_{loop}\Delta t + \frac{\dot{m}}{2}C_pT_b\Delta t - \dot{m}C_pT_v\Delta t - U_vA_v(T_v - T_j)\Delta t \qquad \text{(S12)}$$

where T_b = Temperature of the back-purge stream.

Simplifying and solving equation 12 further gives an equation similar to the energy balance equation 8.

$$\frac{dT_v}{dt} = \frac{\dot{m}\left(\frac{T_{loop}}{2} + \frac{T_b}{2} - T_v\right)}{m} - \frac{U_vA_v(T_v - T_j)}{mC_p} \qquad \text{(S13)}$$

Assuming steady state i.e. $\frac{dT_v}{dt} = 0$ solving and simplifying further gives the equation:

$$T_v = \frac{\left(T_{j_v}\left(\frac{U_v A_v}{\rho Q_t C_p}\right) + \left(\frac{T_b}{2} + \frac{T_{loop}}{2}\right)\right)}{1 + \left(\frac{U_v A_v}{\rho Q_t C_p}\right)} \tag{S14}$$

2.4 Energy balance equation for tube-in-tube heating

The auto-sampling loop uses a tube-in-tube heating system to heat the solution to ensure under-saturated conditions are maintained. To derive the energy balance equation of each tube segment we will need to consider a cross-section of tube with length L as shown in Figure S5. Counter-current flow is occurring between the two streams where the sampling tube inlet temperature changes from T_{in} (cold) to T_{out} (less cold) at velocity v and jacket temperature T_{jin} hot to T_{jout} less hot at velocity v_j .

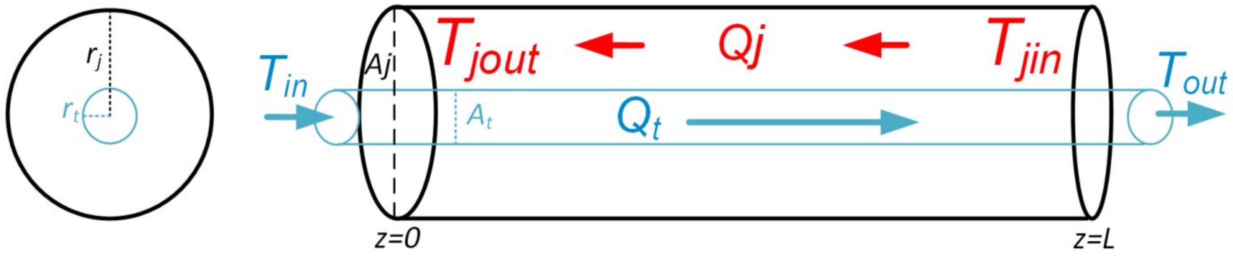

Figure S5. Tube-in-tube heating sampling loop segment of length L showing a schematic of a single tube segment of loop setup with counter-current heat exchange between heating tube segment of area A_j flowing at rate Q_j and sampling tube segment of area A_t flowing at rate Q_t.

To determine the inner tube temperature T at any point down the tube we will begin with the energy balance equation as follows

Accumulation = In - Out + HT(heat transfer)

$$A_t \Delta z \rho C_p (T_{t+\Delta t} - T_t) = \dot{m} C_p \Delta t [T_z - T]_{z+\Delta z} - U 2\pi r_t \Delta z (T - T_j)\Delta t \tag{S15}$$

Dividing by $A_t \Delta z \rho C_p$ and the mass flow rate $\dot{m} = \rho Q$, and solving further gives

$$\frac{\partial T}{\partial t} = -\frac{Q_t \partial T}{A_t \partial z} - \frac{2U\pi r_t (T - T_j)}{\rho C_p A_t} \tag{S16}$$

Equation 16 is derived for the sampling tube. Two equations for counter-current flow are derived from the equation 15 with boundary conditions.

For sampling tube

$$\frac{\partial T}{\partial t} = -\frac{Q_t \partial T}{A_t \partial z} - \frac{2U\pi r_t (T - T_j)}{\rho C_p A_t} \tag{S17}$$

Boundary conditions

$T[z = 0, t] = T_{in}$

$T[z = L, t] = T_{out}$

Initial condition - $T[z, t=0] = T_{start}$

For heated tube in tube jacket

$$\frac{\partial T_j}{\partial t} = \frac{Q_j}{(A_j - A_t)} \frac{\partial T_j}{\partial z} + \frac{2U\pi r_t (T - T_j)}{\rho C_p (A_j - A_t)} \tag{S18}$$

Boundary conditions

$T_j[z = 0, t] = T_{jout}$

$T_j[z = L, t] = T_{jin}$

Initial condition - $T_j[z, t = 0] = T_{jstart}$

For steady state conditions $\frac{\partial T}{\partial t}, \frac{\partial T_j}{\partial t} = 0$. Solving both equations S17, S18 and simplifying further gives us the final equation.

$$\frac{\partial T}{\partial z} - \frac{\partial T_j}{\partial z} = (T - T_j)\frac{-U\pi D_t}{\rho C_p}\left(\frac{1}{Q_t} - \frac{1}{Q_j}\right) \tag{S19}$$

Define $T - T_j = X$ and $B = \frac{-U\pi D_t}{\rho C_p}\left(\frac{1}{Q_t} - \frac{1}{Q_j}\right)$ equation 5 can be written as

$$\frac{dX}{dz} = XB \quad \text{(S20)}$$

Integrating with boundary conditions upper and lower limit

$$\int_{X_{in}}^{X_{out}} \frac{dX}{X} = \int_{0}^{L} B dz \quad (21)$$

$$\frac{X_{out}}{X_{in}} = exp(BL) \quad (22)$$

where for counter-current tube-in-tube flow

$$X_{out} = T_{out} - T_{jin} \text{ at } z = L \quad \text{(22a)}$$

$$X_{in} = T_{in} - T_{jout} \text{ at } z = 0 \quad \text{(22b)}$$

In a similar manner, equations for co-current flow can be derived for the case of liquid sampling.

2.5 Energy balance equation for multiple loop segments of sampling setup

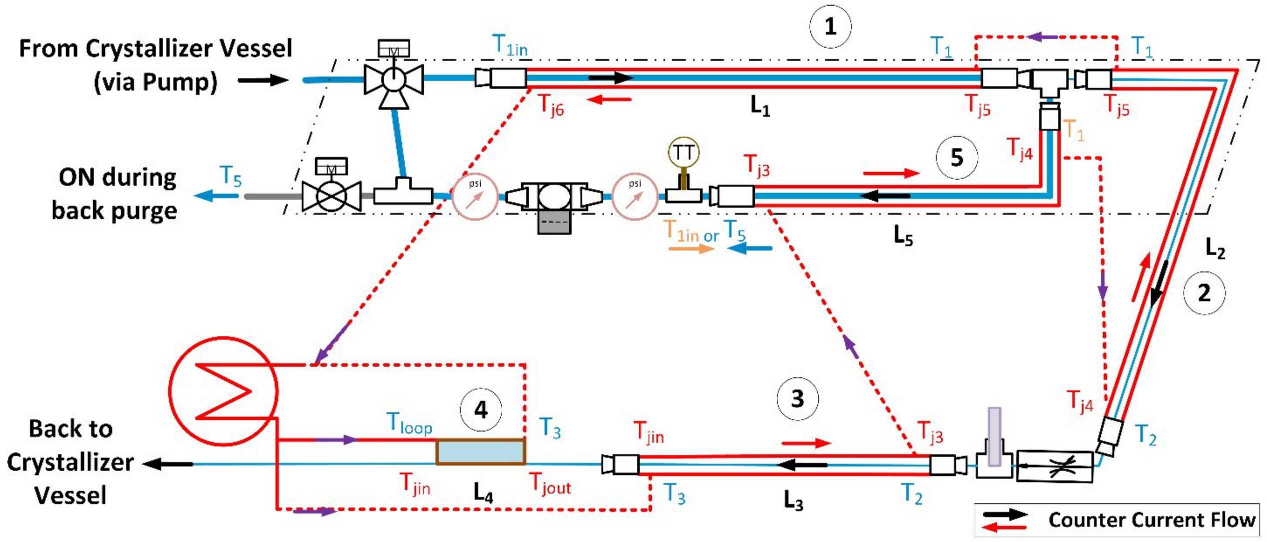

Figure S6. Complete automated sampling setup that consists of crystallizer vessel, sampling loop segments 1, 2, 3, 4, 5 connected with thermostat in series with the inline measurement devices (ATR-FTIR and Polarimeter)

As the sampling loop consists of multiple heating tube-in-tube segments, for which we must derive equations for each segment. Different segments of the loop are shown in Figure S6. The complete setup consists five segments and a crystallizer vessel segment A.

The overall energy balance equation will be derived based on the mode of operation. Consider first the **suspension sampling mode of operation**. The flow sequence initiates sampling from crystallizer A to loop segments 1 → 2 → 3 → 4 and then back to A(vessel).

Along with the suspension sampling back-purge of heated solution in segment 5 is occurring to purge the filter. Therefore segment 5 equation will also be considered. The segment A energy balance equation for the vessel has already been derived in the previous section 2.1. For tube segment 1 of the sampling loop, gives us the simplified energy balance equation:

$$\frac{X_{out}}{X_{in}} = exp(B_1 L_1) \tag{23}$$

Where L_1 = length of the tube segment 1, terms in equation 23 of loop segment 1 according to the schematics figure S6 represent

$$T_v - T_{j6} = \Delta T\,(\text{left}) \text{ or } X_{in} \tag{23a}$$

$$T_1 - T_{j_5} = \Delta T(right) \text{ or } X_{out} \tag{23b}$$

$$\boldsymbol{B_1} = \frac{-\boldsymbol{U\pi D_{t_1}}}{\boldsymbol{\rho C_p}}\left(\frac{\mathbf{1}}{\boldsymbol{Q_{t_1}}} - \frac{\mathbf{1}}{\boldsymbol{Q_{j_1}}}\right) \tag{23c}$$

Putting the values in equation 23 gives us the energy balance for segment 1

$$\frac{T_1 - T_{j_5}}{T_v - T_{j_6}} = exp(L_1 B_1) \tag{24}$$

Similar equations for loop segments 2, 3 and 4 can be derived. Measuring the cell heating jacket of segment 4 and other loop segments are connected in series with the same thermostat so the energy is conserved. As discussed previously, segment 5 involves back-purge and flow between tubes is counter-current as for the other segments. It can be rewritten as

$$\frac{T_5 - T_{j_3}}{T_1 - T_{j_4}} = exp(L_5 B_5) \tag{25}$$

Where L_i = length of tube segments,

$$B_i = \frac{-U\pi D_{t_i}}{\rho C_p}\left(\frac{1}{Q_{t_i}} - \frac{1}{Q_{j_i}}\right) \tag{25a}$$

$i = 1,2,3,4,5$

As tube segment 2, 3 and 1 and 5 have the same inner sampling tube diameters therefore we can write $D_{t2} = D_{t3}$ and $D_{t1} = D_{t5}$. This can help us to consider the flow rate among tubes with the same inner tube diameter to be the same as the overall flow rate across the loop is maintained. For

segment 2, 3 it can be simplified as $Q_{t2} = Q_{t3}$, $Q_{j2} = Q_{j3}$ because $D_{t2} = D_{t3} \rightarrow B_2 = B_3$. However, in the case of back-purge flow in segment 5 during suspension sampling the flow from loop segment 1 splits into segment 2 and segment 5. We consider the simplest case that during the split the flow rate divides into equal amounts (synchronized by pump rpm speed and flow controller). $Q_{t1} = Q_{t2} + Q_{t5}$, $Q_{j1} = Q_{j5}$ and $D_{t1} = D_{t5}$. The energy balance equation of the crystallizer vessel and this model equation 22 can be solved together to determine the overall heat transfer across the loop and the loop temperature operating limitations at a specific flow rate.

2.6 Energy balance for liquid sampling mode

Similarly, for the liquid sampling mode of operation the flow sequence changes starting from the crystallizer to filler loop segment 5 → 2 → 3 → 4 and then back into the crystallizer vessel A (shown in figure S6). During liquid sampling, no flow is occurring through segment 1 so it can be eliminated. However, in segment 5 the heated tube and sample heat flow is parallel instead of counter-current. The segment 5 equation 22 can be rewritten as

$$\frac{T_1 - T_{j_4}}{T_v - T_{j_3}} = exp(L_5 B_{b_5}) \quad (26)$$

The following energy balance equation of the crystallizer equation (11) will be used (opting out the back-purge stream effect)

$$\boldsymbol{T_v = T_{j_v} + \frac{\rho Q C_p}{U_v A_v}(T_{loop} - T_v)}$$

Where $B_i = \frac{-U\pi D_{t_i}}{\rho C_p}\left(\frac{1}{Q_{t_i}} - \frac{1}{Q_{j_i}}\right)$ and L_i = length of tube segment, i = 1, 2, 3, 4, 5 type of loop segment. For co current flow → $B_{b_5} = \frac{-U\pi D_{t_5}}{\rho C_p}\left(\frac{1}{Q_{t_5}} + \frac{1}{Q_{j_5}}\right)$

Combining the loop temperature segment equation according to the mode of operation and integrating it further with population and mass balance model provides the suspension dissolution operating limit in the loop (Section 4.4). PBE model for the loop to describe evolution of particle size distributions, PSD, and the effect of dissolution rate on PSD is critical for which a PBE and MBE model is developed for the heated loop setup.

2.7 PROCESS CONTROL TOOLBOX

Batch reactor vessel controlled with Atlas reactor software system to enable recipe control, real time data plotting. Control box graphic user interface (GUI) was self-designed using **LabVIEW program** shown in Figure S7. It provides a user friendly interface for monitoring and automated control of valve operation modes, sample flow rate, temperature set point values and displays and records real-time measurement of optical rotation, temperature gradient in loop and pressure drop across filter.

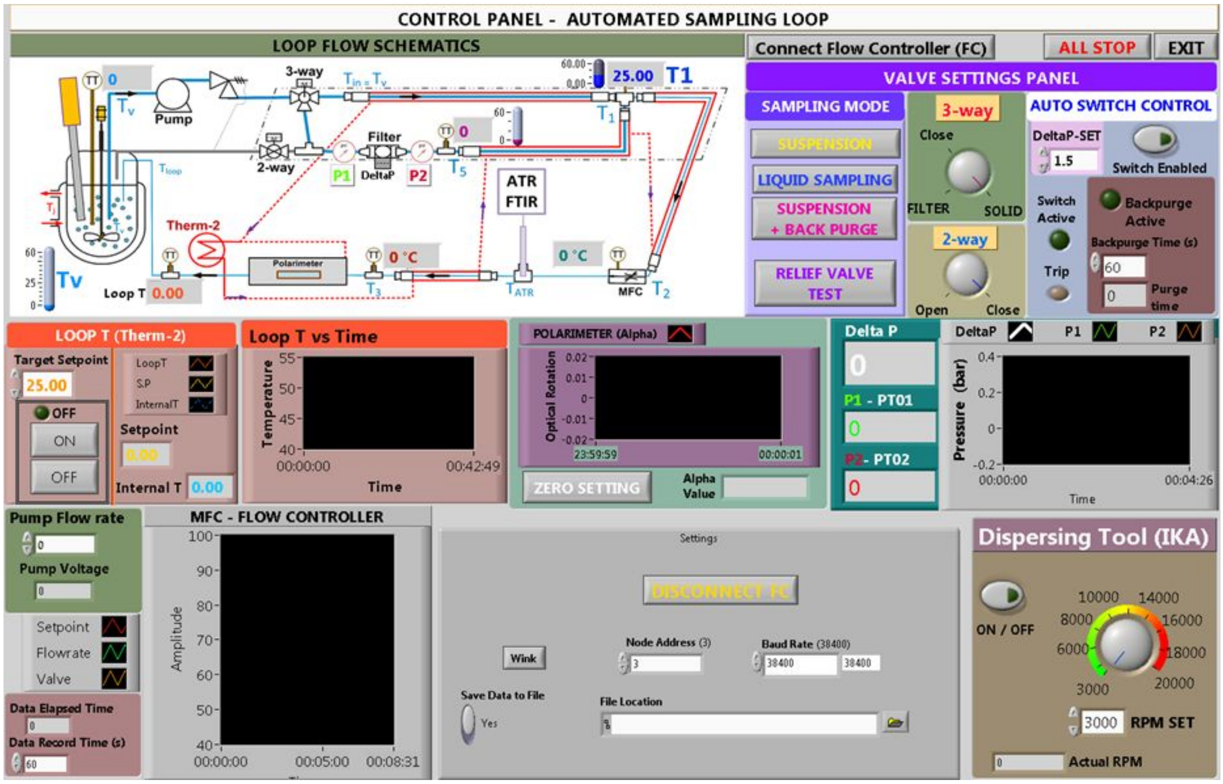

Figure S7 Control panel – LabView program for automated loop setup for online controlling and monitoring all the integrated electronic sensors, valve systems in one system.

2.8 REFERENCES

(1) Cornel, J.; Mazzotti, M. Quantitative Application of in Situ ATR-FTIR and Raman

Spectroscopy to a Crystallization Process. *Anal. Chem.* **2008**, *80* (23), 9240–9249. https://doi.org/10.1021/ac801606z.

(2) Vetter, T.; Mazzotti, M.; Brozio, J. Slowing the Growth Rate of Ibuprofen Crystals Using the Polymeric Additive Pluronic F127. *Cryst. Growth Des.* **2011**, *11* (9), 3813–3821. https://doi.org/10.1021/cg200352u.

Table for Notations

Following Table S2 lists notations used in the equations above.

Table S2: Notations Table

Name	Symbol	Unit
Loop residence time	τ_{loop}	min^{-1}
Loop Volume	V_{loop}	m^3
Vessel Temperature	T_v	K
Temperature sampling tube	T_i	K
Tube jacket Temperature	T_{ji}	K
Vessel jacket Temperature	T_{jv}	K
Loop Temperature end of loop	T_{loop}	K
Temperature in the start of vessel	T_{in}	K
Temperature going out of vessel	T_{out}	K
Back-purge stream Temperature	T_b	K
Length of Tube segments	L_i	m
Diameter of Tube	D_{ti}	m
Radius of Tube	r_t	m
Mass of solution/sample	m	kg
Density of water	ρ	$\frac{kg}{m^3}$
Area of vessel	A	m^2
Surface Area of vessel	A_v	m^2
Mass Flow rate	$\dot{m}$	$\frac{kg}{s}$

Volumetric Flow rate of tube segment	Q_{ti}	$\frac{m^3}{s}$
Volumetric Flow rate of heating jacket fluid	Q_{ji}	$\frac{m^3}{s}$
Overall Heat transfer coefficient of loop segment	U	$\frac{W}{\text{K m}^2}$
Heat transfer coefficient of vessel	U_v	$\frac{W}{\text{K m}^2}$
Heat Capacity	C_p	$\frac{J}{\text{kgK}}$
Flow velocity	V	$\frac{m^3}{s}$
